# Conformational Dissection of a Viral Intrinsically Disordered Domain Involved in Cellular Transformation

**DOI:** 10.1371/journal.pone.0072760

**Published:** 2013-09-27

**Authors:** María G. Noval, Mariana Gallo, Sebastián Perrone, Andres G. Salvay, Lucía B. Chemes, Gonzalo de Prat-Gay

**Affiliations:** 1 Protein Structure-Function and Engineering Laboratory, Fundación Instituto Leloir and IIBBA- CONICET, Buenos Aires, Argentina; 2 NMR Laboratory, Fundación Instituto Leloir and IIBBA-CONICET, Buenos Aires, Argentina; 3 Institute of Physics of Liquids and Biological Systems, Universidad Nacional de La Plata, La Plata, Argentina; 4 Department of Science and Technology, Universidad Nacional de Quilmes, Bernal, Argentina; Aligarh Muslim University, India

## Abstract

Intrinsic disorder is abundant in viral genomes and provides conformational plasticity to its protein products. In order to gain insight into its structure-function relationships, we carried out a comprehensive analysis of structural propensities within the intrinsically disordered N-terminal domain from the human papillomavirus type-16 E7 oncoprotein (E7N). Two E7N segments located within the conserved CR1 and CR2 regions present transient α-helix structure. The helix in the CR1 region spans residues L8 to L13 and overlaps with the E2F mimic linear motif. The second helix, located within the highly acidic CR2 region, presents a pH-dependent structural transition. At neutral pH the helix spans residues P17 to N29, which include the retinoblastoma tumor suppressor LxCxE binding motif (residues 21–29), while the acidic CKII-PEST region spanning residues E33 to I38 populates polyproline type II (PII) structure. At pH 5.0, the CR2 helix propagates up to residue I38 at the expense of loss of PII due to charge neutralization of acidic residues. Using truncated forms of HPV-16 E7, we confirmed that pH-induced changes in α-helix content are governed by the intrinsically disordered E7N domain. Interestingly, while at both pH the region encompassing the LxCxE motif adopts α-helical structure, the isolated 21–29 fragment including this stretch is unable to populate an α-helix even at high TFE concentrations. Thus, the E7N domain can populate dynamic but discrete structural ensembles by sampling α-helix-coil-PII-ß-sheet structures. This high plasticity may modulate the exposure of linear binding motifs responsible for its multi-target binding properties, leading to interference with key cell signaling pathways and eventually to cellular transformation by the virus.

## Introduction

In 1984, Emil Fisher proposed that proteins must acquire a unique three-dimensional globular structure in order to achieve functionality. This hypothesis was first challenged by the discovery of gene sequences that encoded for unfolded proteins [1] and by the existence of proteins that have more than one minimum energy state in their folding landscapes [2]. These groups of proteins, which are functional but lack a compact, well-defined secondary or tertiary structure in solution, are known as “natively unfolded” or “intrinsically disordered proteins” (IDPs) [3], [4]. After the first examples of IDPs were introduced [5], [6], a high proportion of IDPs or proteins with intrinsically disordered domains (IDDs) were uncovered as increasing genomic information for the different organisms became available. IDPs and IDDs are widespread in nature representing 28% of prokaryotic proteomes, and more than 32% of eukaryotic proteomes [7]. They are involved in crucial biological processes such as signaling, molecular recognition and cell homeostasis, and are associated with human pathologies including cancer, neurodegenerative and cardiovascular diseases, among others [8]. Several algorithms have been developed for predicting intrinsic disorder. Some of them are based on the overall residue hydrophobicity/net charge ratio [9], whereas others are based on energy content estimated from aminoacid composition [10]. However, experimental information about the dynamics of IDPs and their conformational ensembles in solution is required for better understanding structure-function relationships in intrinsic disorder and for improving the accuracy of algorithm predictions.

The most widely used experimental techniques for IDP studies include protease mapping, Far-UV circular dichroism spectroscopy (CD), nuclear magnetic resonance (NMR), In-cell NMR, analytical ultracentrifugation (AUC), fluorescence correlation and vibrational spectroscopy, among others [11]. IDPs and IDDs present hydrodynamic properties that indicate that they are extended in solution and present low levels of consolidated secondary structure, high conformational flexibility and dynamic residual secondary structure ensembles, which are highly sensitive to changes in the local environment such as pH, ionic strength and temperature [2], [3]. It has been proposed that the multiple protein interactions involving IDPs can be mediated by linear motifs, short linear interaction-prone segments [12] often present within disordered regions that undergo disordered to ordered transitions upon binding [13]. Some IDPs present strong conformational tendencies towards the bound conformation in the unbound state, suggesting the presence of local preferences for transient secondary structure elements within binding sites [14].

The proportion of IDDs and IDPs was noticed to be particularly high in viral genomes, where a small number of gene products or their combination is sufficient for completion of the viral life cycle. This increased proportion of disordered regions has been associated with high adaptability and mutation rates, and also with the high structural flexibility that allows interaction with multiple cellular and viral targets conferring a multifunctional nature [15], [16]. Paradigmatic examples are papillomaviruses (PVs), which are small double-stranded DNA viruses with circular genomes of less than 9 kbp and only eight polypeptide products [17]. PVs are medically relevant human pathogens, since persistent infections can lead to different types of cancers, in particular cervical cancer [18]. Since PVs lack the required enzymes for viral genome replication and transcription, they depend on the cell machinery in order to carry out their viral life cycle [19]. In this regard, early proteins E6 and E7 interact with the tumor suppressors p53 and retinoblastoma tumor suppressor (pRb), respectively, leading to their proteasomal degradation [20]. Degradation of pRb mediated by E7 causes the release of the general transcription factor E2F, promoting S phase entry and therefore cell cycle progression [21]. E7 is the main transforming product from PVs [22] and has been reported to interact with a large number of cellular targets affecting multiple cell regulatory pathways [23], [24]. These features highlight the binding promiscuity of the E7 oncoprotein, and may explain the many different mechanisms through which E7 can lead to cell transformation and cancer [25].

**Figure 1:**
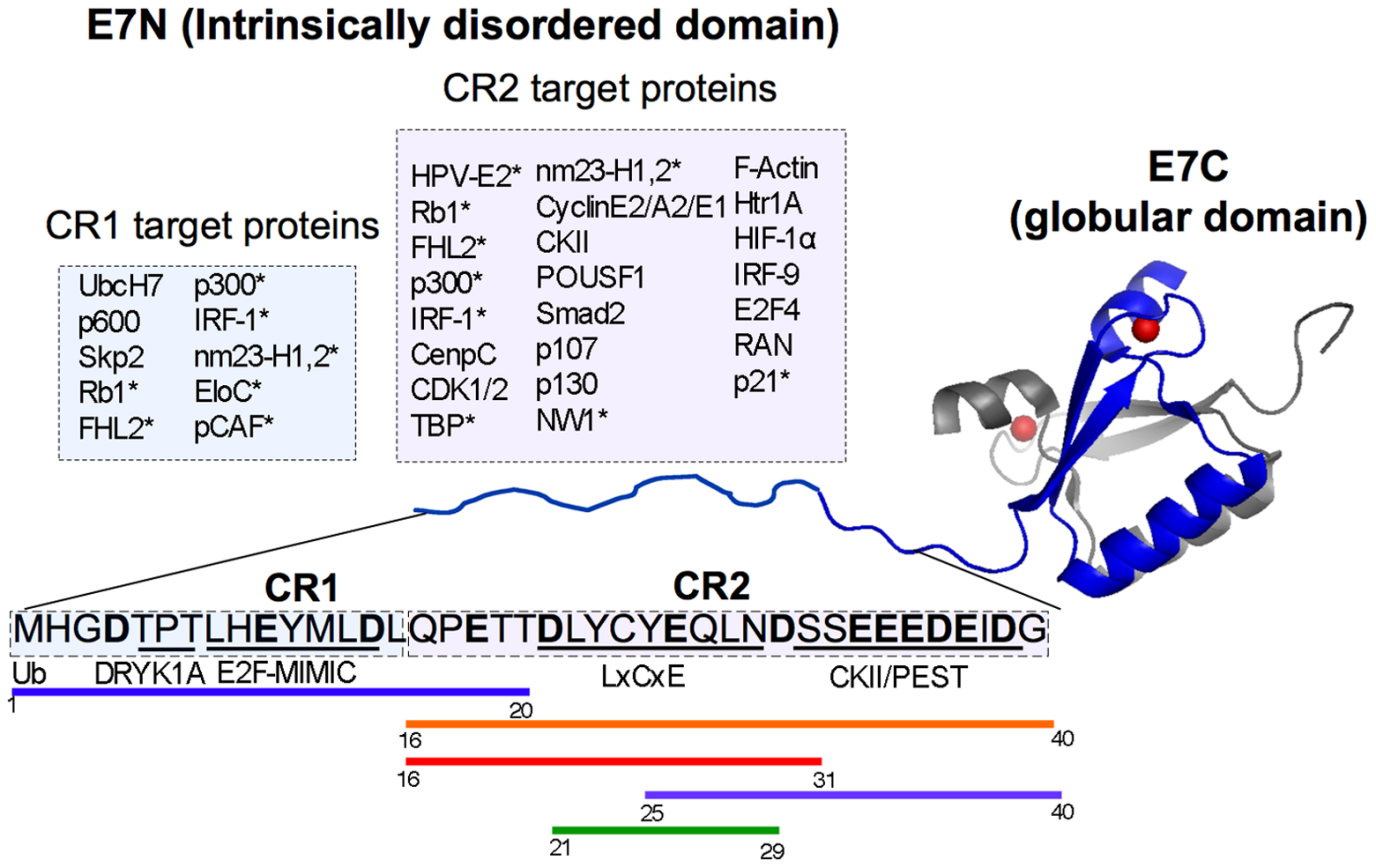
Schematic representation of HPV-16 E7 oncoprotein and the E7N Intrinsically disordered domain. NMR structure of the dimeric HPV-45 E7C domain (PDB ID: 2F8B) with each monomer represented in blue and grey respectively and the structural Zn^2+^ atoms shown as red spheres. The E7N domain from a single E7 monomer is shown as a ribbon with the aminoacidic sequence for HPV-16 detailed in black. Conserved regions CR1 and CR2 are boxed with targets that bind to each region shown in colored boxes above the sequence. (*) Indicates proteins that bind to both conserved regions. The N-terminal ubiquitination site (Ub) and the E7N linear motifs (DYRK1A, E2F-MIMIC, LxCxE, CKII and acidic PEST region) are underlined. The region covered by the peptide fragments used in this work is shown by colored lines below the sequence. E7 (1–20): blue line; E7 (16–40): orange line; E7 (16–31): red line; E7 (25–40): violet line and E7 (21–29): green line.

The human papillomavirus type-16 (HPV16) E7 oncoprotein, is a small, 98-amino acid acidic Zn^2+^ binding protein composed of two domains, the N-terminal domain (E7N, residues 1–40) and the C-terminal domain (E7C, residues 51–98) [26]–[28], which are linked by a proline-rich hinge region [29]. The E7 protein is an extended dimer in solution, which can be described as an IDP [26], [30]. We have shown that the ID nature maps to the E7N region, which was defined as a *bona fide* domain despite the lack of canonical secondary or tertiary structure, or cooperative unfolding [27]. On the other hand, E7C is a dimeric globular domain with two highly conserved CxxC motifs that coordinate zinc [26], [31]. E7N presents two conserved regions, CR1 and CR2, which share sequence alignment and function with two related oncoproteins from small DNA tumor viruses (DNATV), E1A from adenovirus [32], and the large T antigen from SV40 [33]. Six linear motifs have been found within E7N, which could potentially interact with at least 40 cellular and viral targets [24]: an ubiquitination site, the E2F-mimic motif and DRYK1A phosphorylation sites are located within the CR1 region, whereas the LxCxE Rb-binding motif, two phosphorylation sites (S31 and S32 in HIV16 E7) and an acidic stretch containing a PEST degradation sequence are located in CR2 (Figure 1). The remarkable functional conservation among DNATV proteins is highlighted by the presence of the E2F-mimic motif in the E1A CR1 region as well as by the occurrence of the LxCxE Rb-binding motif and the CKII-acidic stretch in the E1A and SV40 large T antigen CR2 regions [34]. In addition, the LxCxE motif is present and highly conserved in several cellular proteins and different DNA and RNA viruses [35], [36]. The E7 LxCxE and CKII-PEST motifs are found close in sequence in most PV E7 proteins, are functionally coupled, and evolved in a coordinate manner [37]. However, a striking example of plasticity of linear motifs within PVs is given by the CKII-PEST motif from bovine papillomavirus type-1 (BPV-1), since this motif is absent from E7 but present in the E2 master regulator protein [38].

We have shown that HPV16 E7 can self-assemble into defined spherical oligomers (E7SOs) upon removal of its coordinated zinc atoms [39]. The E7N domain faces the solvent in E7SOs, providing solubility to the otherwise insoluble E7C oligomer [39], [40]. The highly stable E7SOs present amyloid-like properties, display chaperone holdase-like activity [39], [41] and were shown to be located in the cytosol of HPV-transformed cell lines and cancerous tissue, where they can interact with numerous binding partners [25]. Moreover, the E7 oncoprotein is under the repressive control of the PV E2 master regulator [21], whose open reading frame is disrupted upon integration of the viral genome to the host chromosome. In the absence of E2, E7 levels become deregulated, producing an increase of oligomeric states in the cytosol that may contribute to cellular transformation. The interaction between E2 and E7 takes place through the IDD E7N domain and suggests a mutually sequestering mechanism mediated by oligomerization or aggregation that may balance repression or over expression of E7 [42].

Given the medical relevance of HPV and the prototypic nature of the HPV E7 oncoprotein as a model viral IDP [30], [43], we set out to experimentally dissect transient secondary structure elements within the E7N domain by using a fragmentation approach combined with Far-UV CD and NMR spectroscopies, AUC, and solvent stabilization. We identified two sequence stretches with strong propensity towards α-helical structure, located within the CR1 and CR2 regions respectively. The helix within CR1 is not affected by changes in pH, while the C-terminal region of the helix within CR2 alternates with polyproline type II (PII) structure depending on charge neutralization of the highly acidic stretch. Many of the structures acquired by E7 fragments depend on their sequence context and map to protein interaction sites, highlighting their structural plasticity and functional relevance. We discuss our results in the light of the promiscuous binding properties of the E7N domain and on their possible effect on the local conformational equilibria of the CKII-PEST region, which modulates protein turnover.

## Results

### Dissection of E7 N-terminal domain conformational propensities by fragmentation and solvent stabilization

In order to dissect the structural elements within the intrinsically disordered E7N domain, we recombinantly expressed or synthesized a series of proteins and peptide fragments spanning different regions of the HPV-16 E7N domain and the E7 protein. The sequences of E7N and its sub-fragments are listed and shown in Table 1 and Figure 1, respectively: i) E7 (1–40) comprises the entire E7N domain and contains both the CR1 and CR2 regions; ii) E7 (1–20) spans the CR1 region, which includes the ubiquitination site, the E2F-mimic and the DYRK1A linear motifs; iii) E7 (16–40) covers the entire CR2 region, including the LxCxE pRb binding motif, and the acidic CKII-PEST region; iv) E7 (16–31) covers the CR2 region, excluding the CKII-PEST region; v) E7 (21–29) covers the minimal LxCxE sequence used in the co-crystal with pRb and in NMR studies [44], [45]; vi) E7 (25–40) interrupts the LxCxE motif and includes the acidic CKII-PEST region. Finally, E7 (1–98) corresponds to the dimeric full-length E7 protein and E7 (27–98) to a fragment including the globular dimerization domain of E7 excluding the CR1 and part of the CR2 regions containing the LxCxE motif (Figure 1).

**Table 1:** Aminoacidic sequences of E7N sub-fragments used in this work.

E7 fragment	Aminoacidic sequence^a^
E7N (E7 (1–40))	MHG**D**TPTLHEYML**D**LQP**E**TT**D**LYCY**E**QLNDSS**EEEDE**I**D**G
CR1 (E7 (1–20))	MHG**D**TPTLH**E**YML**D**LQP**E**TT
CR2(E7 (16–40))	QP**E**TT**D** LYCY**E**QLN **D** SS**EEEDE**I**D**G
E7 (16–31)	QP**E**TT**D** LYCY**E**QLN **D**S
E7 (21–29)	**D** LYCY**E**QLN
E7 (25–40)	Y**E**QLN **D** SS**EEEDE**I**D**G

a Acidic residues are shown in bold and the E2F-mimic, LxCxE and CKII/PEST motifs are underlined.

**Figure 2:**
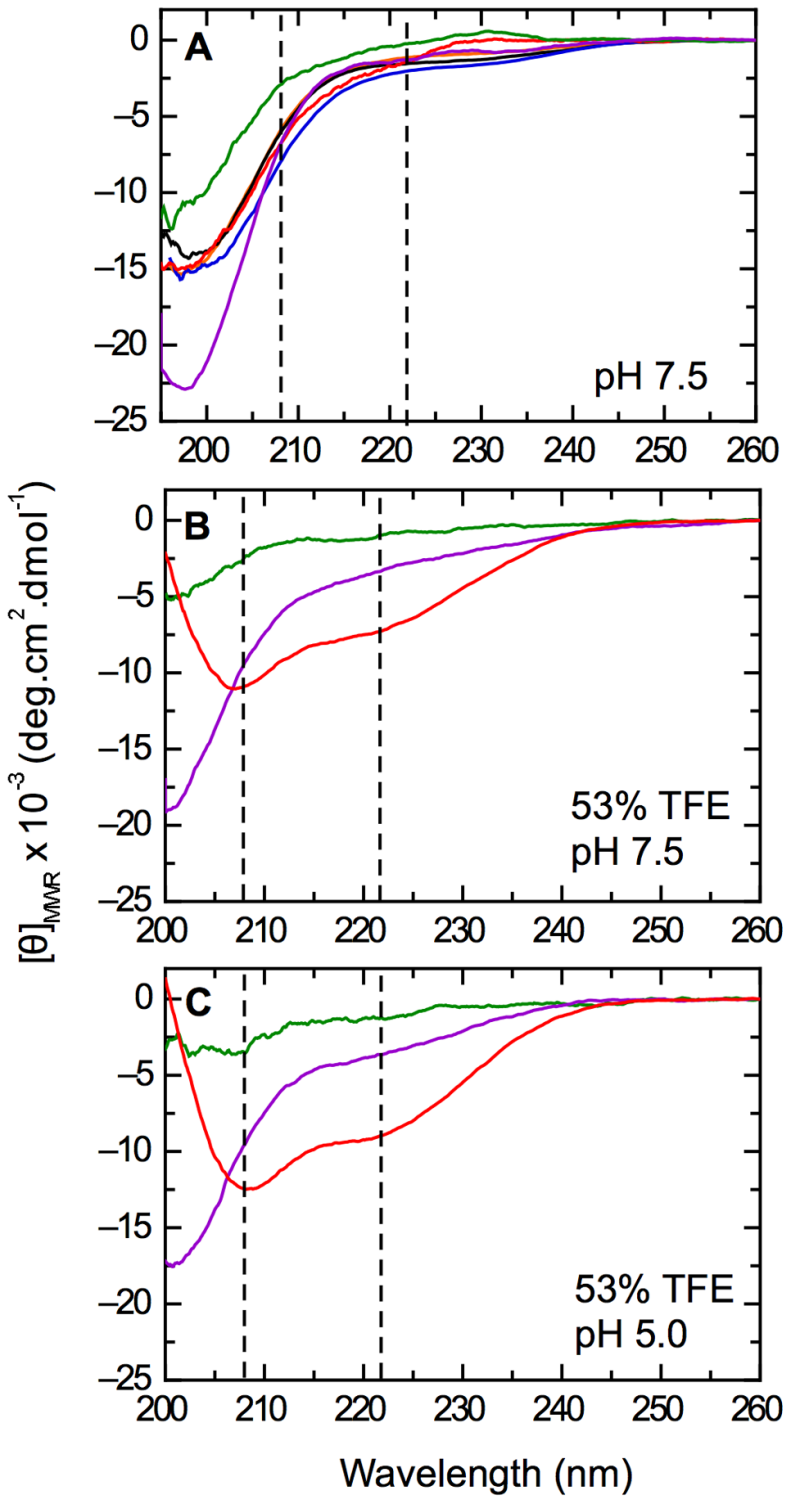
Dissection of transient secondary structure elements within E7N. A) Far-UV CD spectrum of E7 (1–40) and its sub-fragments in 10 mM Tris.Cl pH 7.5 buffer at 20°C. Shown are E7 (1–40): black line; E7 (1–20): blue line; E7 (16–40): orange line; E7 (16–31): red line; E7 (21–29): green line and E7 (25–40): violet line. B–C) Stabilization of pre-existent α-helix structure with 53% TFE for fragments E7 (16–31), E7 (25–40) and E7 (21–29) measured at 20°C in 10 mM Tris.Cl buffer at pH 7.5 (B) and 10 mM sodium formate buffer at pH 5.0 (C). The vertical dotted lines indicate 208 nm and 222 nm wavelengths and the fragment coloring code is as in (A).

Comparison of the far-UV CD spectra of the E7N sub-fragments in 10 mM Tris.Cl buffer at pH 7.5 showed a lack of canonical secondary structure with a general appearance of disorder (Figure 2A). Most sub-fragments presented similar spectra, with molar ellipticity values around −15,000 deg·cm^2^·dmol^−1^ resembling those of the previously described E7 (1–40) domain [27]. However, two sub-fragments presented significant changes in different regions of their spectra: the minimal LxCxE fragment E7 (21–29) showed an atypical shape with a positive band at ∼230 nm that suggested a turn-type structure, and the E7 (25–40) fragment presented a largely increased negative minimum at ∼200 nm (around −23,000 deg·cm^2^·dmol^−1^) characteristic of PII conformation [46] (Figure 2A), suggesting that not “all disorder” in the fragments was similar.

**Figure 3:**
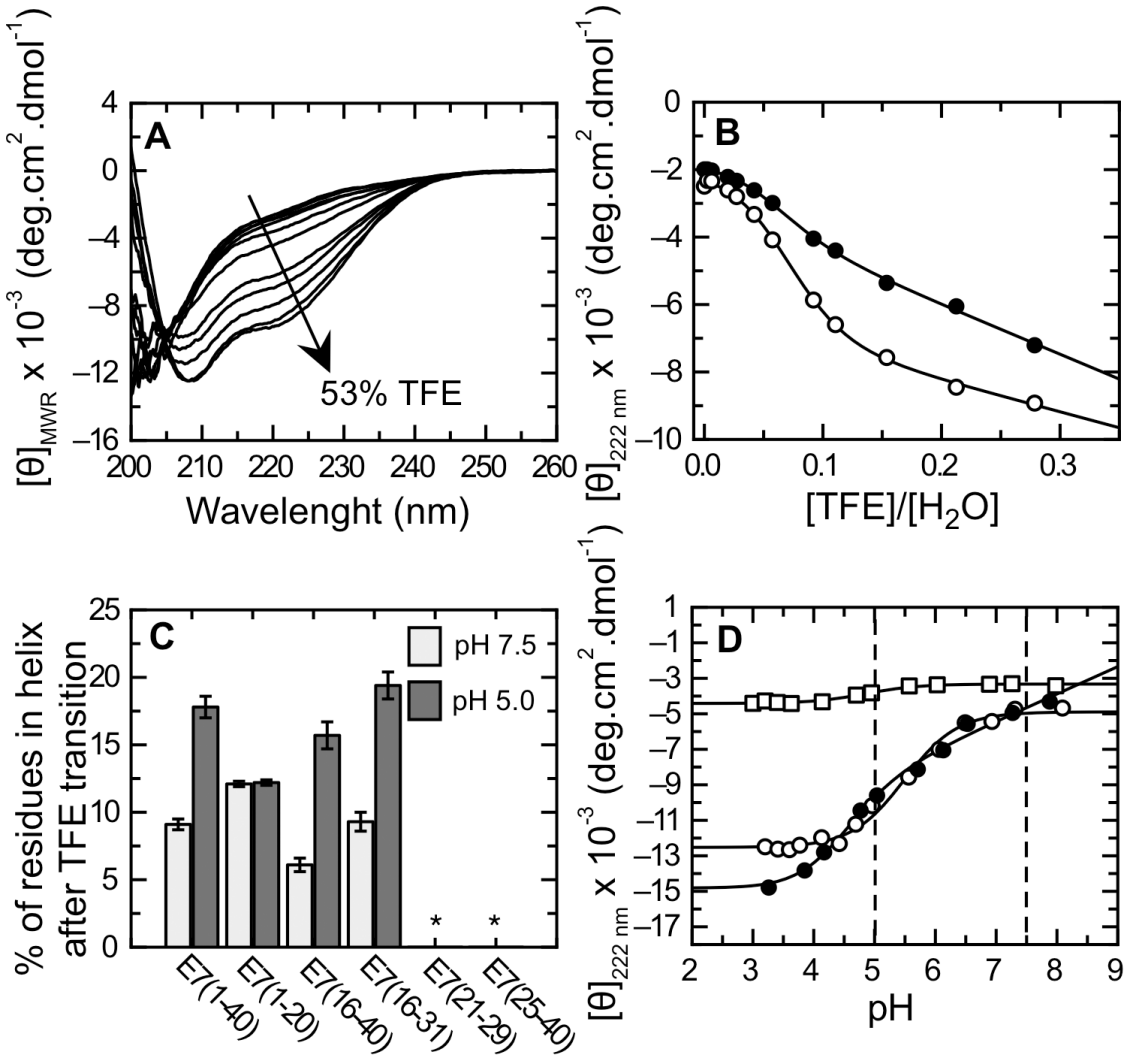
Characterization of α-helical populations within E7N by CD spectroscopy. A) Far-UV CD spectra of E7 (16–31) in 20 mM Tris.Cl buffer pH 5.0 at 20°C and TFE % (v/v) percentages ranging from 0 to 53%. The arrow indicates the sense of change upon increasing TFE. B) Titration curves for E7 (16–31) following ellipticity at 222 nm as a function of [TFE]/[buffer] molar ratio at pH 7.5 (full circles) and 5.0 (open circles). The lines show fitting of the data to a two-state coil-helix equilibrium model (see Materials and Methods and Table 2). C) Maximum percentage of α-helix content induced by TFE in E7N and the sub-fragments using 20 mM Tris.Cl buffer pH7.5 (white bars) and 20 mM sodium formate buffer pH 5.0 (gray bars). The percentage of residues in α-helix conformation was calculated from data fitting of TFE titrations for each fragment to a two-state coil-helix equilibrium model (Figure S1 and Table 2). The symbol (*) indicates no alpha helix induction. D) pH titration curves for different fragments followed by molar ellipticity at 222 nm in 10 mM citrate-phosphate buffer containing 30% TFE. E7 (1–40): dark circles; E7 (16–31): open circles, and E7 (1–20): open squares. Fitting of the data to Equation 4 are plotted as full lines.

Next, we used different solvent mixtures and pH conditions for differentially stabilizing low populated preexistent structures within E7 (1–40). One of the most widely used co-solvents, 2,2,2-trifuoroethanol (TFE), is known to stabilize α-helical populations in sequences with intrinsic α-helical propensity [47]. TFE was earlier shown to stabilize α-helical secondary structure in E7 (1–40), with α-helical content being increased at low pH values [27]. TFE stabilized α-helical structure at pH 7.5 and pH 5.0 in all fragments to different degrees except for the E7 (21–29) and E7 (25–40) fragments (Figure S1). Figures 2B and 2C show an example of stabilization for E7 (16–31) and the lack of helix induction even at 53% TFE for the E7 (21–29) and E7 (25–40) fragments. We performed TFE titrations at pH 7.5 and pH 5.0 for all the sub-fragments. The titrations were visibly cooperative and presented an isodichroic point characteristic of two-state transitions (Figure 3A and Figure S1), leading us to analyze the data according to a two-state coil-helix model (see Materials and Methods)[47]. We show a representative example of the Far-UV CD spectra for TFE titrations of E7 (16–31) at pH 5.0 (Figure 3A) and TFE titrations followed by ellipticity at 222 nm along with data fitting for both pH (Figures 3B), from which the Δ*G^H20^* and *m*-values for helix formation were obtained. The thermodynamic parameters of the E7N sub-fragments were similar to those of the E7N domain (Table 2). The percentage of α-helical populations calculated from the titrations for the E7 (1–40), E7 (1–20), E7 (16–40) and E7 (16–31) fragments was on average below 5% in water (Table 2), and increased to different degrees ranging from 6% to 19% in high TFE (Table 2 and Figure 3C). It must be noted that since the length of the fragments is variable, a higher helical percentage in a small fragment, i.e. E7 (16–31) does not indicate more helical content than a larger one, i.e. E7 (1–40). The maximum percent stabilization of α-helix at high TFE was two-fold larger at pH 5.0 compared to pH 7.5 for the E7 (1–40), E7 (16–40) and E7 (16–31) fragments with a maximum value of 19% (Figure 3C), suggesting a pH-dependent stabilizing effect on helical populations for these fragments. On the other hand, TFE-stabilized α-helical populations in E7 (1–20) to similar degrees at both pH values, with an average value of 12% (Figure 3C). These results suggested the presence of two regions within E7N that could populate α-helical structure and had different pH sensitivity.

**Table 2:** Parameters for α-Helix Stabilization in HPV-16 E7 protein and peptides.

	pH 7.5				pH 5.0			
	*ΔG^H20^* aα-Helix	*m* bTFE	% α-Helix^c^		*ΔG^H20^* aα-Helix	*m* bTFE	% α-Helix^c^	
			H2O	TFE			H2O	TFE
E7 (1–40)	1.2±0.1	23.8±2.7	3.6±0.1	9.1±0.4	1.5±0.2	27.6±3.4	5.7±0.4	17.8±0.8
E7 (1–20)	1.9±0.2	36.4±3.9	5.8±0.2	12.1±0.2	1.4±0.1	33.6±2.4	5.5±0.1	12.2±0.2
E7 (16–40)	0.9±0.2	21.4 ±4.5	2.3±0.4	6.1±0.5	1.4±0.2	23.8±3.9	6.3±0.5	15.7±1.0
E7 (16–31)	1.2±0.2	23.5±4.4	5.7±0.3	9.3±0.7	1.5±0.1	23.5±2.1	6.3±0.4	19.4±1.0
E7 (1–98)	Nd	Nd	10^d^	30^d^	Nd	Nd	16^d^	33^d^
E7 (27–98)	Nd	Nd	9^d^	23^d^	Nd	Nd	8^d^	18^d^

*Nd*: Not determined.

a ΔG is given in kcal/mol.

b m value is given in kcal/mol.

c Percentage of residues in α-helical conformation.

d Values calculated from the initial and final ellipticity values at 222 nm of TFE titrations.

**Figure 4:**
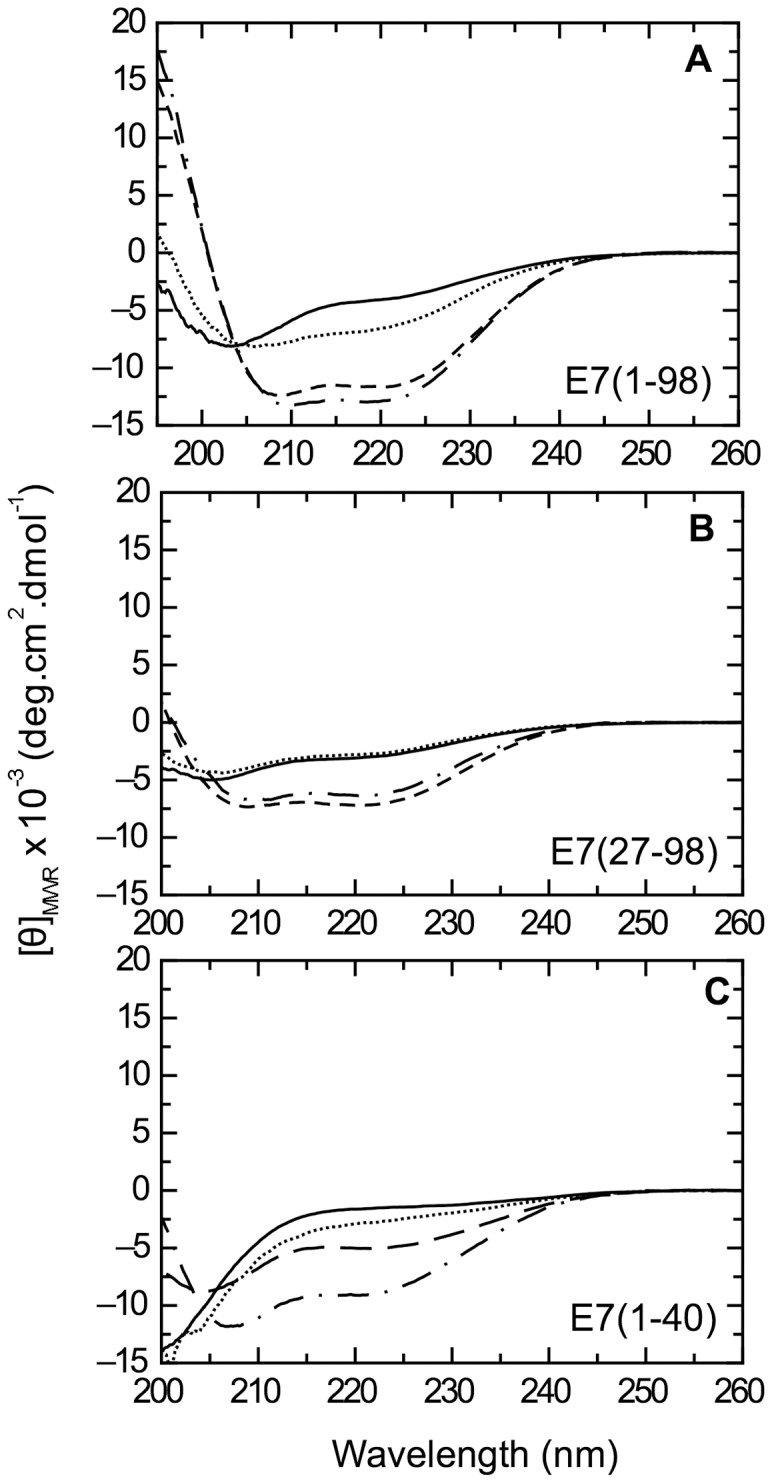
pH dependence α-helix formation in HPV-16 E7, the E7 (27–98) truncated variant and E7N. A–C) Far-UV CD spectra of E7 (1–98) (A), E7 (27–98) (B), and E7 (1–40) (C). Spectra were measured at 20°C in 10 mM Tris.Cl buffer pH 7.5 (full line), 10 mM Tris.Cl buffer pH 7.5 with 53% TFE (broken line), 10 mM sodium formate buffer pH 5.0 (dotted line) and 10 mM sodium formate buffer pH 5.0 with 53% TFE (dotted and dashed line).

In order to assess the influence of the globular E7C domain on the structural determinants of E7N, we analyzed helix stabilization in full-length E7 and in the truncated version E7 (27–98). Far-UV CD spectra of both species are shown in comparison to the E7 (1–40) domain (Figure 4 A–C). While there is virtually no observed change in buffer between pH 7.5 and pH 5.0 for E7 (27–98) (Figure 4B), full-length E7 and E7 (1–40) showed an increase in α-helical content at low pH (Figure 4A, C), suggesting that E7N IDD is the domain that is modulated by pH within E7. Addition of TFE stabilized α-helix to different extents in each species, but only E7N presented a pH-dependent increase in helical content at high TFE (Figure 4C). Given the tendency of E7C to oligomerize in the absence of E7N [40], TFE experiments could not be performed on this isolated domain. However, we will assume that the 27–50 fragment cannot stabilize α-helix because i) the highly acidic E7 (25–40) fragment is not sensitive to TFE addition, and ii) the inter-domain “hinge” region is rich in proline residues [29]. Therefore, we consider that the increase in α-helical content in E7 (27–98) upon TFE addition is due to the stabilization of native α-helix structure in the globular E7C domain [28] (Figure 4B). The molar ellipticity value at 222 nm for full-length E7 in high TFE (around −13,000 deg·cm^2^·dmol^−1^, Figure 4A) corresponds to 30% helical population (Table 2) and roughly to 30 residues in α-helix conformation, which is significantly larger than the α-helical content of E7C (20 residues) [28], further suggesting the induction of helical populations within E7N in this condition.

To further investigate the role of pH on stabilization of α-helix populations within E7N, we carried out pH titrations at 30% TFE (Figure S2), a concentration that is close to saturation for most titrations (Figure S1). E7 (1–40) presented more than one group of titrating residues with an average *pKa* of 4.4±0.2, while E7 (16–31) showed a single transition with an average *pKa* of 5.5±0.1, (Figure 3D) and E7 (16–40) had a *pKa* of 5.0±0.1 (Figure S2). These values were in good agreement with those obtained for solvent exposed aspartic and glutamic residues (3.9 and 4.3 respectively) [48]. The slightly higher values obtained experimentally may be due to contribution of titrating residues with higher *pKa* values (His, Cys or Tyr) or to the decrease in the dielectric constant produced by TFE [49]. Taken together, these results suggested that the sigmoideal pH transitions observed were due to protonation of acidic residues within E7N. The transitions in E7 (1–40), E7 (16–40) and E7 (16–31) could be interpreted as increases in the α-helical content at low pH, evidenced by a shift of the minimum at 200 nm to 208 nm and by a decrease in the band at 222 nm in acidic conditions (Figure S2). E7 (25–40), which did not stabilize α-helix upon addition of TFE (Figure 2 B,C), displayed a clear sigmoideal transition with an average *pKa* value of 5.2±0.1 (Figure S2). However, the CD spectrum of E7 (25–40) at low pH did not show the minima characteristic of α-helix (Figure S2), suggesting that titration of acidic residues in this fragment induced a conformational change that did not correspond to a helix-coil transition. Furthermore, E7 (1–20), which showed α-helix induction that was independent of pH, showed a small transition in pH titrations, confirming that secondary structure in this fragment was not sensitive to charge (Figure 3D). Finally, the minimal E7 (21–29) fragment showed no variations in its CD spectrum as a function of pH (Figure S2).

**Figure 5:**
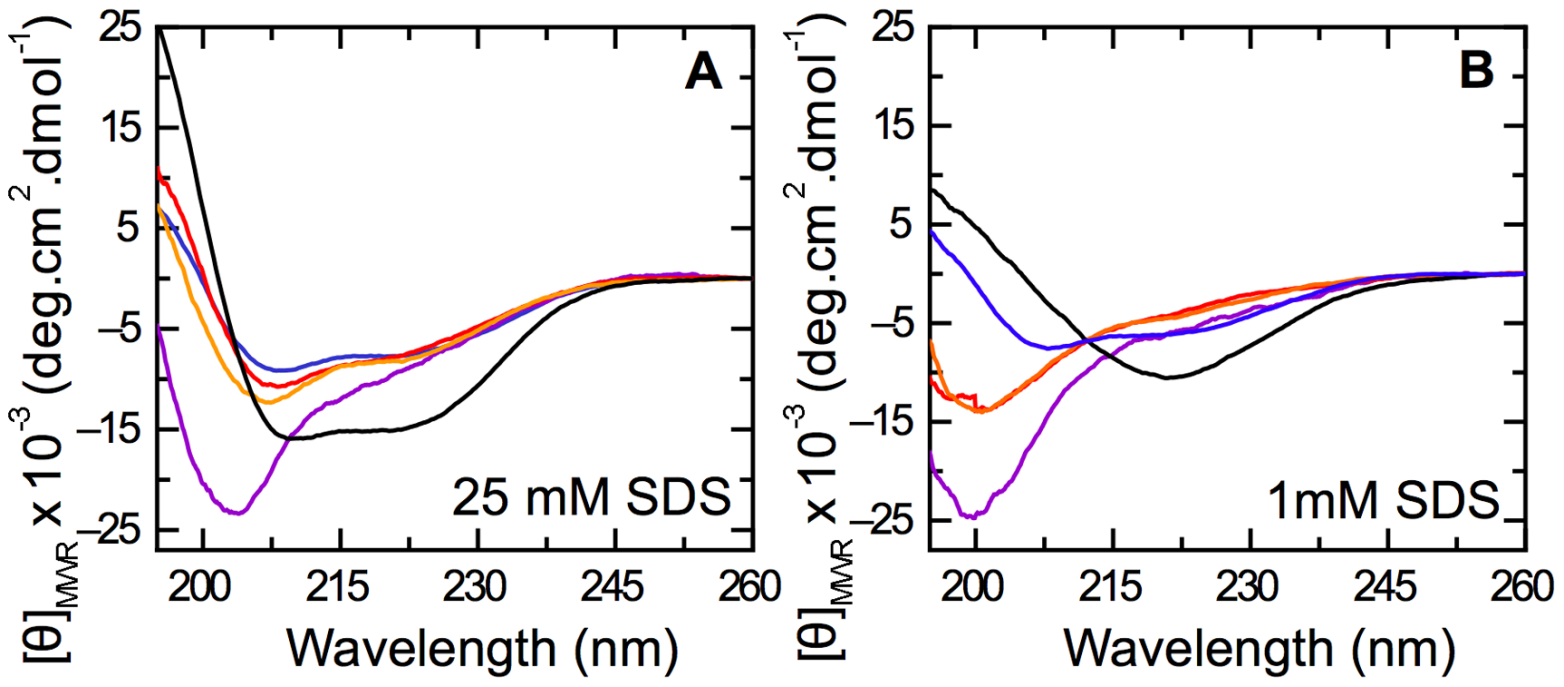
Effect of the anionic detergent SDS on secondary structure of E7N and its fragments. A–B) Far-UV CD spectra of E7 fragments in 10 mM sodium formate buffer pH 3.0 containing 25 mM SDS (A) or 1 mM SDS (B). Fragments are E7 (1–40): black line; E7 (1–20): blue line; E7 (16–40): orange line; E7 (16–31): red line, and E7 (25–40): violet line. The critical micellar concentration (CMC) calculated for SDS in this experimental condition is 5 mM.

A further means of stabilizing local secondary structure in peptides is the anionic detergent sodium dodecyl sulfate (SDS), which is known to stabilize α-helix in E7N at supra-micellar concentrations (CMC  = 5 mM), and may stabilize ß-sheet conformations at sub-micellar concentrations [27]. In order to minimize charge effects, we worked at pH 3.0 where all the acidic residues were neutralized. At 25 mM SDS (supra-micellar concentration), all fragments tested except for E7 (25–40) stabilized α-helix to different extents (Figure 5A), in agreement with the TFE results. At 1 mM SDS (sub-micellar concentration), we found that only E7N showed a tendency to form a ß-sheet structure. However, sub-fragments E7 (16–31), E7 (16–40) and E7 (25–40) showed Far-UV CD spectra similar to those in aqueous buffer, indicating no stabilizing effect of SDS (Figure 5B). The E7 (1–20) fragment stabilized α-helix even at sub-micellar SDS concentrations (Figure 5A–B), which confirmed the high propensity of this fragment towards α-helical populations and was in line with the results from TFE experiments, where high helical content was induced irrespective of pH (Figure 3D). Overall, these results confirmed the helical propensities observed in TFE experiments and suggested that the formation of ß-sheet structure within E7N might involve the establishment of long-range interactions.

### Analysis of hydrodynamic properties of E7N fragments

The homogeneity of molecular mass, size and shape was measured at pH 7.5 using analytical ultracentrifugation (AUC) sedimentation velocity experiments [50] for selected fragments including the entire E7N domain and the CR1 and CR2 regions (E7 (1–40), E7 (1–20) and E7 (16–40), respectively). All peptides showed homogeneous sedimentation profiles and produced best fits to a single species model, allowing estimations of their hydrodynamic parameters and apparent molecular weight (*M_app_*) (Figure S3 and Table 3). The estimated *M_app_* values agreed within experimental error with the theoretical mass (*M_theorical_*) of all peptides, indicating that all fragments were monomeric in solution (Table 3). Peptides showed no propensity to self-association at pH 7.5, and concentration dependence experiments revealed the presence of weak repulsive inter-particle interactions for peptides spanning all E7N regions (Figure S4), which could be due to repulsion between charged acidic residues at neutral pH.

**Table 3:** Hydrodynamic properties of E7 (1–40), E7 (1–20) and E7 (16–40).

	*S*_0_^20, w^(S)	*D_0app_* ^20, w^(10^−7^ cm^2^ s^−1^)	*M_app_*(Da)	*M_theorical_*(Da)	(*f/f_min_*)^a^	*R_H_ (AUC)* b(Å)	*R_H_ (NMR)* b(Å)	*R_H_ (NMR)* c(Å)
E7 (1–40)	0.77±0.01	12.5±0.1	4900±200	4678	1.53±0.01	17±1	15.3±0.2	14.3±0.3
E7 (1–20)	0.50±0.01	17.6±0.5	2400±200	2330	1.40±0.02	12±1	Nd	Nd
E7 (16–40)	0.71±0.01	15.9±0.6	3400±200	2923	1.45±0.03	13±1	Nd	Nd

*Nd*: Not determined.

a Mean value obtained from c(s) analysis.

b *R_H_* measured at pH 7.5.

c *R_H_* measured at pH 5.0.

We estimated the frictional ratio of the peptides, *f/f_min_*, which is a useful parameter for describing the shape of macromolecules in solution [50] (see Materials and Methods). The *f/f*_min_ values of globular proteins are nearly constant, increasing from 1.15 to 1.3 for the 5- to 1000 kDa molecular mass range, while *f/f*_min_ values for IDPs are significantly larger and increase from 1.5 to 3 for molecular mass ranging from 5 to 200 kDa [3], [50]. The *f/f_min_* values for all E7N fragments had values ranging between 1.40 and 1.53 (Table 3), which indicated that both the E7N domain as well as the E7 (1–20) and E7 (16–40) fragments had hydrodynamic properties characteristic of IDPs. Finally, we analyzed the hydrodynamic radius (R_H_) for the different fragments by using AUC at pH 7.5 and for the E7N domain by pulsed field gradient NMR experiments (PFG-NMR) at pH 7.5 and pH 5.0. PFG-NMR measures molecular diffusion rates and accurately complements the overall analysis of size and shape of proteins in solution by AUC. The R_H_ values for the E7N domain from AUC and NMR measurements at pH 7.5 and similar concentrations (∼ 1mM) were 17±1 Å and 15.3±0.2 Å, respectively. The small discrepancy of the R_H_ values obtained may be due to differences intrinsic to both techniques. Both R_H_ values obtained for E7N were significantly larger than the R_H_ value expected for a 40 residue globular protein (13.8 Å) [51], confirming the IDP nature of E7N. However, both values were also smaller than the R_H_ value predicted for a fully unfolded polymer (18.1 Å) [51], which suggested some degree of compaction of the domain that may be due to the presence of transient long-range interactions. PFG-NMR experiments showed a further decrease in the R_H_ value at pH 5.0 (14.3±0.3 Å, Table 3), suggesting that protonation of the acidic residues contributed to E7N compaction.

### Chemical shift assignment of poorly dispersed E7N spectra

**Figure 6:**
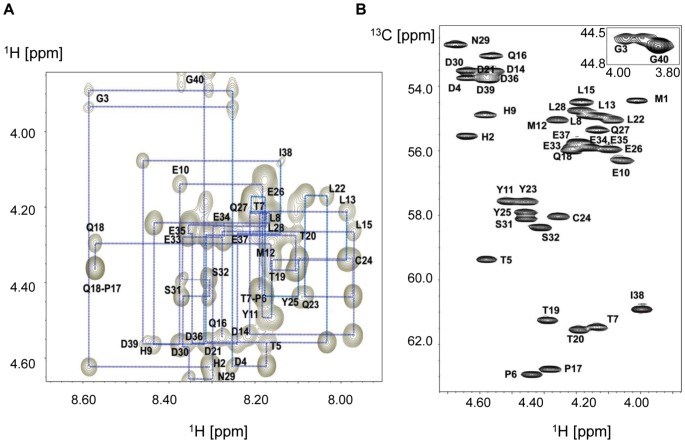
E7N chemical shifts assignments. A) Finger-print region of 2D ^1^H-^1^H NOESY spectrum of E7 (1–40) in aqueous solution at pH 5.0, showing sequential NOE connectivity among H_α_ nuclei. For clarity, only NH-H_α_ (i, i) and NH-H_α_ (i+1, i) cross peaks are indicated in the figure. B) Selected region of the ^1^H-^13^C HMQC spectrum of E7 (1–40) in aqueous solution at pH 5.0. The assignment of ^13^C_α_-^1^H_α_ cross-peaks is shown. The inset shows the glycine assignment.

An essential step towards the description of fluctuating conformational equilibria in IDPs is the complete assignment of residue chemical shifts by NMR. A first inspection of the one dimensional ^1^H spectrum of E7N in aqueous solution at pH 5.0 showed poorly dispersed amide proton resonances (data not shown), with most peaks within the 7.8–8.5 ppm range, making assignment a rather challenging task. The methyl proton region also showed poor dispersion of peaks, confirming the expected disordered nature of E7N. Using two-dimensional ^1^H-^1^H and ^1^H-^13^C spectroscopy we were able to fully assign the proton and carbon resonances of E7N in aqueous solution at pH 5.0 (Figure 6 and BMRB code: 19269). Due to the great signal overlap in the amide proton region, the ^1^H-^13^C HMQC-TOCSY spectrum was essential to assign the different spin systems, while the sequential assignment of E7N was based on NOE connectivity in the ^1^H-^1^H NOESY spectrum (Figure 6A). In the ^1^H-^13^C HMQC spectrum, residue resonances were mainly grouped by amino acid type, a feature also characteristic of IDPs (Figure 6B).

Interestingly, in all the experimental conditions under which we studied E7N (see below), both prolines, P6 and P17, were fully in *trans* configuration. Only one set of peaks was observed for each proline residue and, as determined from ^13^C_β_ and ^13^C_γ_ chemical shifts [52], they corresponded to the *trans* conformation. In addition, in the NOESY spectrum, the diagnostic strong H_δ_-H_α_ (i, i-1) NOEs for the *trans*-proline isomer was observed for P6 and P17 residues, while no H_α_-H_α_ (i, i-1) cross peak, characteristic for the *cis*-proline conformation, was detected for either proline residue (data not shown). This is in contrast with the linker region joining the disordered N-terminal and the globular C-terminal domains of E7, which also harbors two proline residues (P41 and P47), that present a *cis-trans* isomeric equilibrium [29].

### Transiently populated α-helical conformations by NMR chemical shift analysis

**Figure 7:**
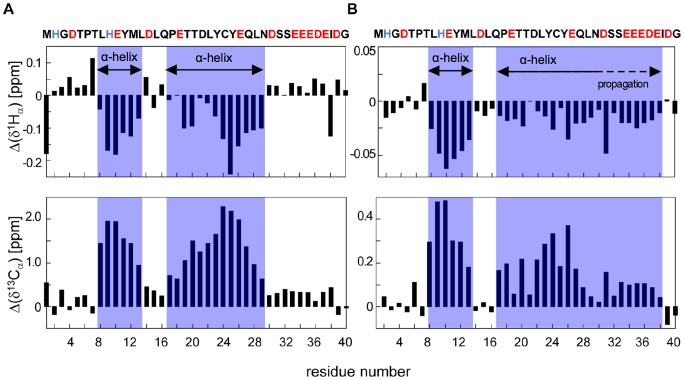
Determination of transient α-helixes within E7N by chemical shifts analysis. ^1^H_α_ and ^13^C_α_, corresponds to chemical shift differences between aqueous and aqueous-TFE solutions for 3 mM E7 (1–40) in 50% TFE-*d*_2_ at pH 7.5 (A) and in 12%TFE-*d*_2_ at pH 5.0 (B). TFE content differs at both pHs, leading to different magnitude of the chemical shifts differences at pH 7.5 and pH 5.0. Regions with transient α-helical conformation are shaded in blue. The E7 (1–40) aminoacidic sequence is shown in the upper part of the figure with acidic residues shown in red and histidines in blue.

Secondary chemical shifts are normally used to assess both the location and population of secondary structure elements in proteins. However, secondary structure in IDPs is typically transient and confined to short individual helical or extended segments with ensemble-averaged structured populations that can range a few percent. Because chemical shifts represent a population weighted average of local conformational preferences, secondary shifts for IDPs are generally small and consequently, secondary transient structures are difficult to detect [53]. Frequently, uncertainties in the random coil shifts can lead to ambiguities in this type of analysis of IDPs [54]. Short range NOEs can be also used to corroborate secondary structure propensities detected through chemical shifts, although in our case this was not possible because of the significant signal superposition in the amide and alpha regions of the proton spectrum. As a consequence, in order to estimate the E7N secondary structure propensities, we compared the chemical shifts of the domain under pH and solvent polarity conditions that stabilize particular conformations of the peptide as revealed by Far-UV CD experiments (with and without TFE at pH 5.0 and 7.5). The presence of TFE caused significant chemical shift changes and line broadening in the ^1^H spectrum, suggesting that the peptide underwent a major structural change in this condition, in agreement with the results from Far-UV CD experiments. At pH 7.5 and 3 mM E7N (the concentration required for NMR assignments at ^13^C natural abundance), we were able to measure chemical shifts using 50% TFE, where maximal helical content is reached (Figure 7A and ) in spite of the fact that the spectra quality was poorer due to signal broadening (data not shown). In contrast, at pH 5.0 we observed E7N precipitation at 3 mM peptide and high TFE concentrations, and for this reason we assigned the resonances at 12% TFE, where the α-helical stabilization is still significant (Figure 7B and Figure S1) and no precipitation was detected. Thus, we fully assigned E7N in the four experimental conditions: pH 7.5 and pH 5.0, in the presence or absence of TFE (BMRB code: 19269 and Tables S1–S3). Although the structure of E7N in TFE: water mixtures was also largely disordered, an increase in chemical shift dispersion indicated a higher degree of stabilization of secondary structure elements. The ^13^C_α_ and ^1^H_α_ shifts are the most robust indicators of residual secondary structures [53]. Positive differences of ^13^C_α_ chemical shifts in TFE solutions with respect to water are an indication of α-helical propensity for a given segment of residues, while negative differences in ^1^H_α_ in TFE with respect to water indicate propensity for α-helical conformation. The chemical shift data at pH 7.5 (Figure 7A) clearly highlight consecutive negative ^1^H_α_ and positive ^13^C_α_ chemical shift differences for regions encompassing residues ^8^LHEYML^13^ and ^17^PETTDLYCYEQLN^29^, indicating the presence of two distinct regions with α-helical conformation within E7N. The similar Δ*G^H20^* and *m*-values obtained for the helix-coil transition in fragments E7 (1–20) and E7 (16–40) which contain these regions (Table 2) suggests that both helixes have similar thermodynamic stability. Figure 7B shows the analysis of chemical shift differences at pH 5.0. Because of the different TFE contents, the magnitude of the shifts at pH 5.0 and pH 7.5 are not comparable. However, the same segments that display α-helical preferences at pH 7.5 also do so at pH 5.0. In addition, the second helix is propagated to the C-terminal stretch of the domain, comprising the acidic CKII-PEST region and spanning from residue P17 to I38 as judged by ^13^C_α_ and ^1^H_α_ chemical shifts. Despite the fact that shifts suggest that the helical structure in this segment is less populated than the N-terminal region of the helix, this acidic region is able to partially stabilize the α-helical conformation at low pH. The NMR experiments showed that a total of 20 residues are involved in α-helix at pH 7.5 and 50% TFE. In contrast Far-UV CD TFE titrations yielded values of ∼10% residues in α-helix conformation in this condition (Table 2) which corresponds to an average of 4 residues fully populating the α-helical conformation [55]. This value was five-fold lower than the number of residues identified by NMR, suggesting that α-helical segments within E7N were partially populated, with average values of about 20%, and highlighting the dynamic nature of these conformations.

### Analysis of E7N polyproline type II elements by Far-UV CD and coupling constants

**Figure 8:**
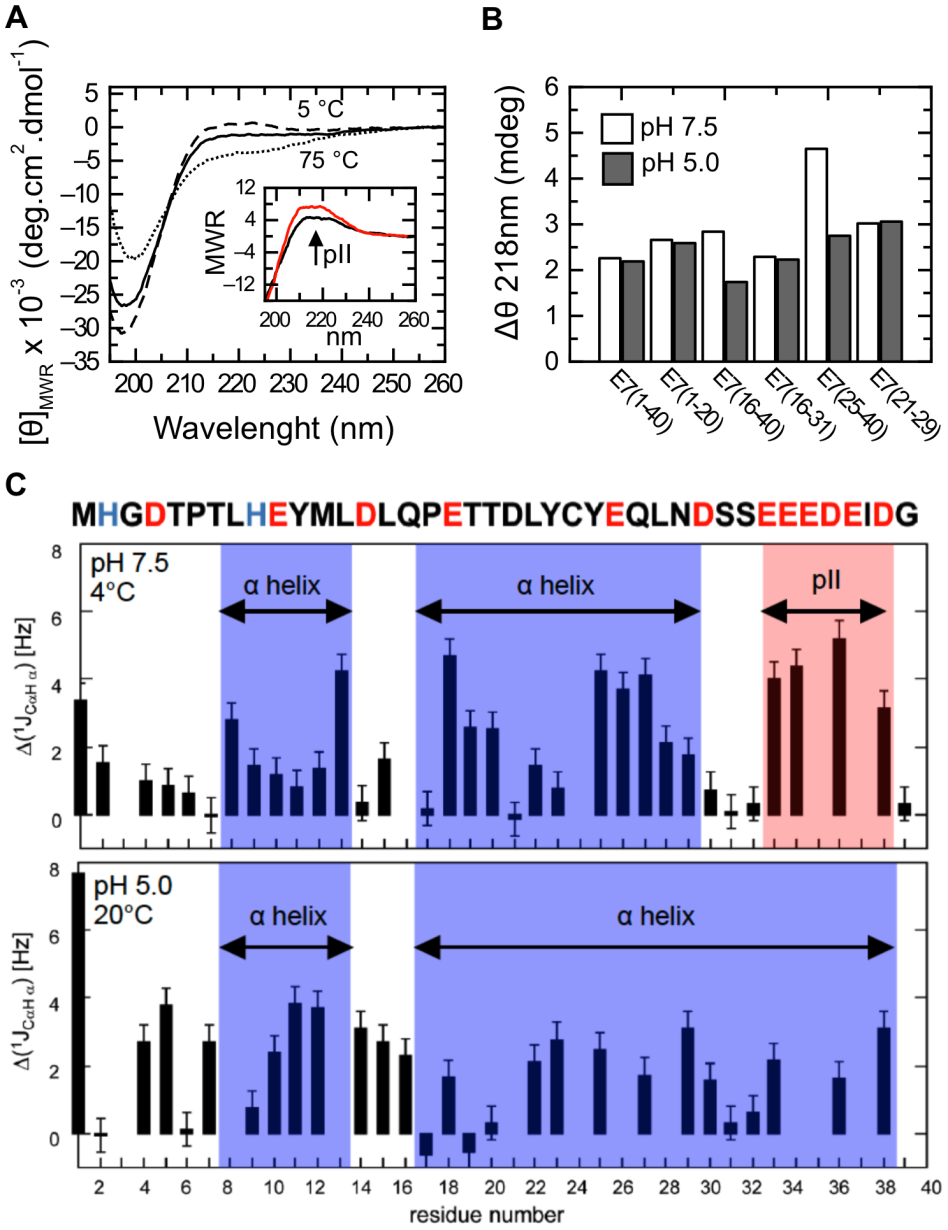
Polyproline type II propensity within E7N. A) Far-UV CD spectra of E7 (25–40) in 10 mM buffer Tris.Cl pH 7.5 at 5°C (broken line), 25°C (full line) and 75°C (dotted line). Inset: Difference spectrum between 5°C and 75°C (black line) and between pH 8.0 to pH 3.0 at 30% TFE for the E7 (25–40) fragment (red line; data taken from CD spectra in Figure 2). The arrow shows the positive band at 218 nm characteristic of PII structure. B) Molar ellipticity at 218 nm (Δθ218) obtained from the difference spectrum between 5°C and 75°C for each E7 peptide. White bars: 10 mM buffer Tris.Cl pH 7.5. Dark bars: 10mM buffer sodium formate pH 5.0. C) Plot of the differences between observed and random coil ^1^JC_α_H_α_ values vs. the position along the E7N sequence, for aqueous solution pH 7.5 at 4°C (upper panel) and for aqueous solution pH 5.0 at 20°C (lower panel). Regions that populate α-helical and PII conformations in each pH condition are shaded in blue and red, respectively. The E7N amino acid sequence is shown in the upper part of the figure with acidic residues and histidines shown in red and blue respectively.

Polyproline type II structure is prevalent in unfolded or intrinsically disordered proteins [56]. However, this conformation is not trivial to identify as it can be stabilized only locally at the individual residue level, and can present features characteristic of disordered chains, often confused with “random coil” properties [56]. Detailed analysis of Far-UV CD spectra can be used to detect PII structure. The intensity of the minimum typical of “disordered” states at ∼198–200 nm and of the positive band at 218–220 nm are sensitive probes for measurement of PII content [46]. Moreover, low temperature stabilizes PII conformations leading to a decrease in the minimum at 198 nm and to an increase in the positive band at 218 nm [56]. In order to study the regions within E7N that can adopt PII structure, we analyzed the Far-UV CD spectra of all the E7N sub-fragments at different temperatures (Figure S5). Figure 8A shows this analysis for the isolated E7 (25–40) fragment. Low temperature led to a decrease in ellipticity at ∼200 nm and to an increase in ellipticity at ∼218 nm (Figure 8A). The difference spectrum (5°C –75°C) showed the induction of a strong positive band at 218 nm and a concomitant decrease at ∼200 nm, suggesting that PII structure was prevalent within E7(25–40) (Figure 8A inset). This was in agreement with the results from pH titration of this fragment, which also showed a difference spectrum (pH 8.0– pH 3.0) suggestive of the induction of PII structure (Figure 8A inset). Further analysis of the difference spectra for all sub-fragments revealed an increase in PII content at pH 7.5 with respect to pH 5.0 only in the E7 (25–40) and E7 (16–40) fragments (Figure 8B), which suggested that deprotonation of the polyacidic stretch at neutral pH led to an increase in charge repulsion that favored an extended PII structure. This was a strong indication that the poly-acidic CKII-PEST region was enriched in PII structure, as we previously described for the inter-domain CKII-PEST containing peptide of BPV-E2 [38].

We aimed at obtaining further evidence of PII structure within E7N by complementing the secondary chemical shifts study with the analysis of ^1^J_CαHα_ couplings by NMR [57]. Given that observed scalar couplings are population weighted averages of couplings sampled over various conformations, any deviation from random coil values can be interpreted as a secondary coupling contribution in analogy to secondary chemical shifts. In particular, ^1^J_CαHα_ coupling constants are a reliable indicator of both α-helical and PII structures at the residue level. Regions populating PII structure are difficult to identify by NMR because the PII backbone conformation does not exhibit characteristic proton or carbon chemical shift deviations from random coil values as found in α-helix and β-sheet conformations [58]. A characteristic pattern of short-range NOE connectivity is also distinctive of PII conformations [59]. Nonetheless, the number of observable inter-residue correlations in the E7N NOESY spectra was limited and precluded the use of NOE connectivity to identify PII regions. A good indication of PII structures is also provided by ^1^J_CαHα_ constants, which present values larger than random coil values [60]. However, α-helices also display increased ^1^J_CαHα_ constant values. Therefore, regions that do not present chemical shift deviations but exhibit relatively large ^1^J_CαHα_ values are candidates to populate PII conformations.

In order to have further confirmation of α-helical propensities and to investigate the presence of PII structure in E7N, we measured ^1^J_CαHα_ values, obtained from a proton coupled HMQC spectrum (Table S4). Figure 8C shows the difference between observed and random coil ^1^J_CαHα_ values [61], Δ^ 1^J_CαHα_ for pH 5.0 and pH 7.5. The deviations of Δ^ 1^J_CαHα_ values were in agreement with the preference for α-helical conformation of ^7^TLHEYML^13^ and ^17^PETTDLYCYEQLND^30^ tracts at both pH values, as determined from chemical shift analysis (Figure 7). In water at pH 5.0 and 20°C, the constants displayed positive deviations from H9 to Q16 and from L22 to I38 for residues whose constants could be obtained, providing an indication of residual α-helical structure in these regions. The slight discrepancies between information derived from chemical shifts and coupling constants may be due to the use of constant random coil values that do not take into account neighbor and side chain charge effects [61]. We further measured the ^1^J_CαHα_ constants and Δ^ 1^J_CαHα_ values in aqueous buffer at pH 7.5 and at low temperature (4°C) where PII conformation is stabilized. Despite the large peak overlapping, we were able to measure four coupling constants in the PEST region (E32, E33, D36 and I38, Figure 8C), which presented positive differences respect to tabulated random coil values. The fact that at pH 7.5 the PEST region exhibits positive Δ^ 1^J_CαHα_ values (Figure 8C) yet no significant chemical shift dispersion even in the presence of TFE (Figure 7A), makes it a candidate for PII stabilization. Taken together, the NMR results strongly suggest that the ^33^EEEDEI^38^ tract presents PII conformation at pH 7.5. Finally, both CD and NMR experiments indicate that when acidic side chains in the E7 (25–40) region are deprotonated at pH 7.5, the electrostatic repulsion stabilizes extended conformations such as PII and precludes the formation of α-helix. Conversely, at low pH where acidic side chains are neutralized, α-helix conformations are favored.

## Discussion

In the present work we performed a structural dissection of the HPV-16 E7N IDD [27] by making use of a fragmentation approach in combination with spectroscopic and biophysical techniques. This domain belongs to a prototypical viral oncoprotein, which is considered as the main transforming protein from PVs and shares sequence similarity and functional properties with related viral oncoproteins. Moreover, the E7N IDD contains several linear recognition motifs located in its CR1 and CR2 regions that are responsible for its multi-target binding properties [37].

Most sub-fragments used for the structural dissection display similar molar ellipticity values, disordered-like Far-UV CD spectra with a minimum at ∼200 nm, and present transient α-helical populations that can be stabilized by TFE to different degrees (Figure 2 and 3). Two fragments within CR2 show no α-helix propensity, even at high TFE, and display distinct features in their spectra: E7 (21–29) comprising the LxCxE motif presents a spectrum typical of a turn-type structure, while E7 (25–40) including the CKII-PEST region displays a drastically increased negative ellipticity at 198 nm, pointing to a PII-type structure within this segment [56], [62]. In addition, AUC experiments showed that E7N (E7(1–40)), the CR1 (E7(1–20)) and CR2 (E7(16–40)) fragments are monomeric and extended in solution, with similar *f/f_min_* values characteristic of IDPs. These results show that although E7N and its conserved regions present global hydrodynamic properties typical of IDPs, not “all disorder” within the E7N domain is similar.

**Figure 9:**
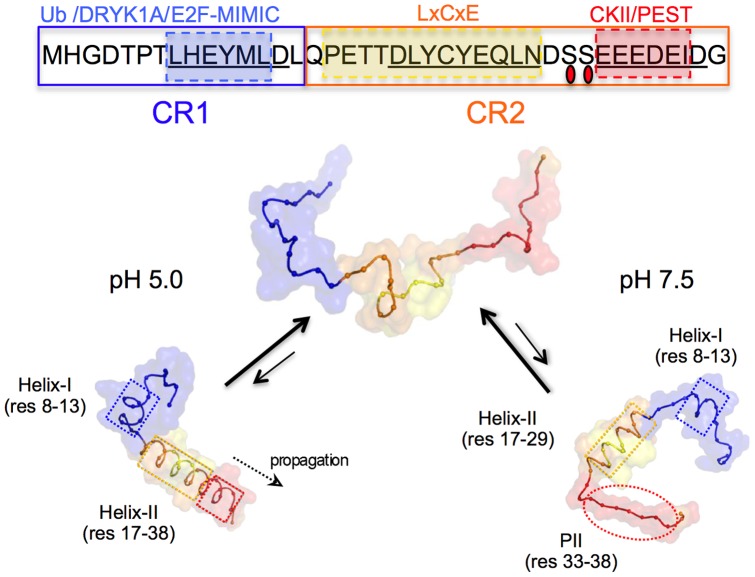
Model of E7N conformational ensembles at pH 7.5 and pH 5.0. In the upper part of the figure the E7N sequence is shown with CR1 and CR2 regions boxed in blue and orange, respectively. The Ubiquitination site (Ub), and the DRYK1A, E2F-MIMIC, LxCxE Rb-binding and CKII/PEST motifs are displayed above the sequence. Phosphoserine residues are indicated with red ovals and regions that stabilize α-helix and PII by punctuated rectangular boxes. The lower part of the figure shows snapshots indicating the particular elements of secondary structure that are populated by the E7N conformational ensemble at pH7.5 and pH5.0 with arrows indicating conformational equilibria and partial population of secondary structure elements. E7N regions show the same color-coding as the upper panel, with the location of helixes and PII structure indicated by boxes and ovals respectively.

We found a pH-dependent increase in α-helical content within E7 (1–40) at high TFE that was not observed in either full-length E7 or in the truncated version E7 (27–98) (Figure 4), which indicates that the information required for pH-dependent α-helix formation is local and lies within the E7N domain. By performing Far-UV CD and NMR measurements, we identified three regions with propensity to populate transient secondary structural elements: two α-helixes located within the CR1 and CR2 regions, respectively and a region rich in PII structure within CR2 (Figures 6–8). NMR studies revealed that at pH 7.5 the α-helixes spanned residues L8 to L13 (Helix I) within CR1 and P17 to N29 (Helix II) within CR2, while residues E33 to E37 within the acidic stretch present PII conformation. At pH 5.0, Helix I remains unchanged, while Helix II propagates into the acidic region, encompassing residues P17 to D38 (Figure 9). Far-UV CD measurements of the E7 (25–40) fragment support these results, showing a decrease in PII structure at low pH, which is presumably due to a reduced electrostatic repulsion between the acidic residues that relaxes the extended conformation and favors the propagation of the α-helix into this region. This is similar to what is observed in poly-glutamic sequences, which also preferentially populate PII conformational space when glutamate side chains become deprotonated [63]. These results reveal a conformational switch between PII structure and α-helix within the CR2 acidic stretch, which is triggered by charge neutralization at low pH.

Interestingly, fragment E7 (21–29) comprised within Helix II is not able to stabilize an α-helix in isolation even at high TFE (Figure 2), which suggests that the residue required for helix initiation is not contained within this peptide [47]. NMR experiments show that P17 is the first residue of Helix II in E7N, in agreement with the fact that the minimal region capable of forming an α-helix is E7 (16–31), which contains P17. These results, together with previous evidence showing that proline is frequently found as an N-capping residue in helixes [64] lead us to hypothesize that P17 may act as the initiation site for Helix II in E7N. In addition, the high conservation of P17 within HPV E7 proteins [37], [43] suggests a functional role for this structural element across HPV E7 proteins. Although further testing will be required to assess this possibility, the compaction of E7N at low pH measured here by PFG NMR and reported by previous size exclusion chromatography experiments [27] suggests that Helix II within E7N may be further stabilized through transient long-range interactions between the E7 CR1 and CR2 regions. In addition, SDS was able to stabilize a ß-sheet type structure in E7 (1–40) that was not observed in any of the E7N sub-fragments (Figure 5), indicating that local sequence information is not sufficient for the formation of this structure within E7N and suggesting the presence of transient long-range interactions, whose nature and extent will need to be addressed by future NMR studies.

Most of the linear motifs identified in E7N are located within the regions that populate transient secondary structures (Figure 9). The ubiquitination site (N-terminus) and DYRK1A phosphorylation sites (residues 5–7) within CR1, which presumably require to be highly accessible for post-translational modification, are located N-terminal to Helix I (residues 8–13). The E2F-mimic motif (residues 8–14) almost exactly matches the location of Helix I, while the Rb binding LxCxE motif (residues 21–29) lies within Helix II. Finally, the CKII sites (S31 and S32) and the acidic stretch (residues 30–40) are located in the region that switches between PII and α-helix structure (∼ residues 30–38). The location of linear motifs in regions of dynamic secondary structure may explain the binding promiscuity of this domain, allowing to switch between alternate conformations and binding to different protein partners. While some regions may be optimized for protein-protein interactions (E2F-mimic and LxCxE motifs), others might expose linear motifs related to post-translational modifications or degradation (ubiquitination, DYRK1A and CKII-PEST sites). The presence of these linear motifs in related DNA tumor virus proteins suggests that the dynamic conformational properties described for E7 may be shared by E1A and SV40 large T-antigen.

Two of the main interaction motifs within E7N show different conformational properties related to their target-bound forms. The E2F-mimic motif forms a α-helix in the bound form [65], [66], while the region corresponding to this motif (residues 8–14) presents high α-helical propensity in the unbound form. This suggests that binding of the E2F-mimic motif to Rb may involve the selection of preformed structural elements [14], although further work will be required to test this hypothesis. On the other hand, the LxCxE Rb-binding motif presents an extended ß-strand like conformation in the bound state [44], [67]. This region presents an extended, turn-like Far-UV CD spectrum in isolation, but is part of transient Helix II in the context of the E7N domain. These different structural tendencies of the LxCxE motif might be largely influenced by the neighboring sequence context, by environmental conditions, or by transient long-range interactions. The population of conformations that do not correspond to the Rb-bound state in this region could constitute an example of functional “misfolding” within IDPs, where conformations populated by the unbound state partially protect binding sites from undesired contacts [68]. Alternatively, the helix conformation may mediate binding to additional targets by this region. Interestingly, kinetic studies of complex formation between the retinoblastoma tumor suppressor AB domain and E7N fragments including E7 (21–29) revealed that if present, the sampling of conformational ensembles in this region is very fast [69] in contrast to the E7N-E7C hinge region, where the interaction mechanism with a specific antibody (M1) displays a slow conformational selection step involving proline isomerization [29]. The different timescales of conformational equilibria within E7N may be associated to a diversity of binding mechanisms, a salient feature of intrinsically disordered domains that may further contribute to their multi-target binding properties.

CKII and PEST sites are contiguous in most HPV E7 proteins, evolved together, and are functionally coupled in Rb binding [24], [37]. Phosphorylation of S31 and S32 within the CKII-PEST region increases Rb binding affinity and PII content in this region [24], [70]. Results from this work show that the presence of negative charge destabilizes Helix II and increases PII content in the CKII-PEST region, which may favor an extended conformation of the LxCxE motif that enhances Rb interaction affinity. The helix-PII equilibrium in this E7N region may also be related to protein degradation. We have previously shown that phosphorylation of a CKII-PEST motif within an intrinsically disordered “hinge” region of BPV-1 E2 that shows the presence of both α-helix and PII separated by a turn leads to an increase of protein turnover *in vivo* [38]. This example introduced a new concept whereby phosphorylation was not used as a “label” for recognition by kinases or the degradation machinery but instead destabilized local structure within this region, causing a decrease in thermodynamic stability that made the CKII-PEST site more accessible for degradation [38]. Although this hypothesis must be further tested, switching between α-helix and PII structure induced by physiological pH variations or phosphorylation might hinder the propagation of the Helix II into the acidic region at pH 7.5, leaving the PEST site more accessible in the form of PII, with direct consequences on E7 turnover.

Interestingly, a very recent publication reported secondary α-helical structures from homology and ab initio modeling in the same regions that we describe here by solvent stabilization and NMR [71]. There is an important difference in that the extent of formation of the HELIX II is largely pH dependent and is in equilibrium with pII structure, something that cannot be predicted by modeling. In any case, the ID domain of HPV-16 E7 samples a discrete number of conformations, but its most stable structure in solution is extended and intrinsically disordered.

In summary E7 (1–40) is a paradigmatic example of an IDD defined through bioinformatic and experimental analyses, which is able to populate dynamic but discrete structural ensembles that are tuned by pH. As the acidic residues are deprotonated, their side chains are electrostatically repelled, and the peptide adopts a more extended conformation involving an increase in PII at the expense of a loss in α-helix. The PV virus life cycle depends on the differentiation from basal epithelial cells to keratinocytes, which may involve substantial changes in the physicochemical environment inside the cell including parameters such as crowding, redox state or pH. Our results suggest that these changes could impact on the conformational equilibria and function of E7 through sampling of α-helix-coil-PII-ß-sheet structures that may modulate the exposure of post-translational modification, degradation and protein interaction sites. This conformational plasticity may potentiate E7 interference with key cell signaling pathways required for completion of the viral life cycle and contribute to cellular transformation by the virus.

## Materials and Methods

### Protein expression and purification

The recombinant E7 HPV-16 protein (E7 (1–98)) was expressed and purified as previously described [26]. The truncated E7 protein E7 (27–98) comprising residues 27 to 98, was cloned as a thrombine cleavable fusion protein to the maltose binding protein (MBP) into a pMALp2 vector (New England Biolabs, Berverly, MA) and expressed in *E. coli* BL21 strain. Cell cultures were grown in 2L of 2TY medium at 37°C containing 0.1 mg/ml ampicillin. The induction of cultures was performed four hours after inoculation by adding 0.4 mM IPTG. Cells were harvested by centrifugation after 12 h of induction. For the purification procedure the protocol described for the full length E7 (1–98) [26] was followed. Protein purity was >95% as judged by SDS/PAGE, and protein identity was confirmed by MALDI-TOF mass spectroscopy (MS) (Bruker Daltonics, Billerica, MA, USA). Protein concentration was determined by the Bradford method using BSA as a standard.

### Peptide synthesis, quality control and quantification

The E7 peptides used in this work, E7 (1–40), E7 (1–20), E7 (16–40), E7 (16–31), E7 (21–29) and E7 (25–40), were synthesized by F-moc chemistry (W.M Keck Facility, Yale University, New Haven, CT) and purified by reverse phase HPLC. The purity of all preparations was judged by MALDI-TOF MS. The peptides were dissolved in Buffer Tris.Cl 50 mM pH 8.0 to a concentration of approximately 1 mM and were stored at −80°C. Quantification was carried out by absorbance at 276 nm in buffer solution and 220 nm in HCl. Peptide sequences are shown in Table 1.

### Buffers and solutions

Unless stated otherwise, measurements at pH 7.5 were performed in 10 mM Tris.Cl buffer and at pH 5.0 in 10 mM Sodium formate buffer, both with 1mM DTT at 20+/−0.1°C. All chemical reagents were of analytical grade (purchased from Sigma Aldrich) and all solutions were prepared with distilled and deionized water (Milli-Q plus) and filtered through 0.22 µm membranes prior to use.

### Far-UV Circular Dichroism (CD) spectroscopy

CD measurements were carried out on a Jasco J-810 spectropolarimeter (Jasco, Japan) with cell paths of 0.1 and 0.2 cm. CD spectra were recorded between 195 and 260 nm at standard sensitivity and at a 50 nm/min of scanning speed with a response time of 8 s, data pitch of 0.1 nm and a bandwidth of 2 nm. All spectra were an average of at least 8 scans. Baseline measurements using buffer alone were subtracted from the measured spectra. Protein concentrations used for the E7 (1–98) and E7 (27–98) were 10 µM. Peptide concentration ranged between 20 to 80 µM depending on the peptide size. Raw data were converted to molar ellipticity, using the following equation:



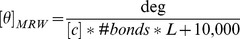
(1)


Where deg is the raw signal in millidegs, [c] is protein concentration in molar units, # bonds is the number of peptide bonds (number of amino acids –1), and L is the path length in cm.

### Analysis of alpha helical content in different solvent mixtures

*2,2,2-trifluoroethanol (TFE) measurements.* TFE stabilization of helical conformations is stronger at lower temperatures, with little variation between 5°C and 25°C [47]. TFE titration curves were carried out at 20°C, a temperature that produces maximal stabilization in model peptides [47]. Samples were dissolved in 20 mM Tris.Cl buffer at pH7.5 and in Sodium formate buffer at pH 5.0 and equilibrated in 0 to 53% TFE (volume of TFE added/total volume added) [62]. *Data analysis.* The mean residue ellipticity at 222 nm, which is assumed to be proportional to helical content, was plotted as a function of [TFE]/[H_2_O] ratio and was fit to a two state “coil to helix” equilibrium model [47]. This model assumes that the free energy for α-helix formation depends linearly on [TFE]/[water] ratio. To extract the thermodynamic parameters Δ*G^0, H20^* and *m*, molar ellipticity data was fitted to the following equation:



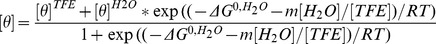
(2)Where [θ]*^H2O^* and [θ]*^TFE^* are the mean residue ellipticities in water and at high TFE concentration, R is the gas constant and T is the temperature in Kelvin. The average percentage of residues in alpha helix conformation in water and TFE for each peptide was calculated from the values [θ]*^H2O^* and [θ]*^TFE^* obtained from the fitting, using the following empirical relationship to define the molar ellipticity value expected for 100% of helix [55]:



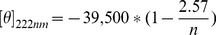
(3)Where n is the number of residues for a given peptide. The TFE titration data from the full length protein E7 (1–98) and the truncated E7 (27–98) protein could not be fitted to the two state model. In this case, the percentage of residues in alpha helical conformation was calculated from the initial and final molar ellipticity values at 222nm.

*pH titration curves at 30% TFE*. The samples were prepared in 10 mM Citrate phosphate buffer ranging from pH 3.0 to 8.0 mixed with 30% TFE and incubated for one hour at room temperature.

*Data analysis.* The mean residue ellipticity at 222 nm as a function of pH was fit to a variant of the Henderson-Hasselbalch equation [72] to extract the global *pKa* value for each fragment, assuming that all ionizable groups are deprotonated in the initial state and protonated in the final state:



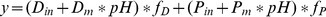
(4)Where y is the measured signal, *Din* and *Pin* are the spectroscopic signals of the deprotonated and protonated states, respectively and *Dm* and *Pm* account for the linear variation of the signals with pH. The fractions of deprotonated and protonated species (*f_D_* and *f _P_*) are defined as:



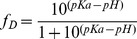
(5)




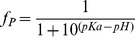
(6)


*Sodium dodecyl sulfate (SDS)*. Experiments were carried out in 10 mM sodium formate buffer at pH 3.0. Samples were equilibrated for one hour at room temperature with different concentrations of SDS (100 µM to 25 mM). The critical micelle concentration (CMC) for sodium formate buffer used in the present work was previously reported [27].

### Analysis of polyproline type II (PII) structure by circular dichroism

Low temperatures were used in order to stabilize PII structures 56]. CD spectra for the different E7 peptides were measured at temperatures ranging from 5 to 75°C. The propensity to adopt PII structure for each peptide was assessed by analyzing the difference spectrum (5°C–75°C) at 218nm [46].

### Analytical ultracentrifugation of peptides

Sedimentation velocity (SV) analytical ulracentrifugation experiments (AUC) were performed for peptides E7 (1–40), E7 (1–20) and E7 (16–40) in 50 mM Tris.Cl buffer at pH 7.5 (Figure S3). All AUC experiments were performed on a Beckman Coulter XL-I analytical ultracentrifuge. SV experiments of solutions were performed at 20°C, at a rotor speed of 50000 rpm using the 8-hole ANTi-50 rotor. Cells were equipped with sapphire windows. Titane double sector centerpieces from Nanolytics Inc. were used. Cells with centerpieces of 0.3 cm optical path were filled with 100 μl of sample and solvent reference. SV profiles were acquired during 24 hours, using absorbance optics, at intervals of 13 min for each cell. Density and viscosity of the buffer, which are required for the analysis, were calculated with SEDNTERP software, from John Philo (http://www.jphilo.mailway.com/). Partial specific volume of peptides was calculated from the amino acid sequences using SEDNTERP. Analyses of SV experiments were made using the continuous distribution c(s) and the non-interacting species model analysis of SEDFIT software from P. Schuck (http://www.analyticalultracentrifugation.com). In order to study non-ideality effects in solution, various protein concentrations were assessed (dilutions 1∶1, 1∶2 and 1∶3). The sedimentation and diffusion coefficients *s_0_* and *D_0app_* were derived from linear approximations to infinite dilution (Figure S4) and used to obtain the apparent molecular mass (*M_app_*) and hydrodynamic radius (*R_H_*) for each peptide according to the Stokes-Einstein and Svedberg equations:



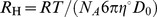
(7)

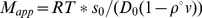
(8)Where *R* is the gas constant, *T* the absolute temperature, *N*_A_ Avogadro's number and *η* solvent viscosity (see Ref. [50] for a complete description of the methods). The *f/f_min_* value depends on the hydration, surface roughness, shape and flexibility of the particle. This value is the ratio of the hydrodynamic radius (*R_H_*), which depends on the size of the molecule in solution, to the minimum theoretical hydrodynamic radius of anhydrous volume (*R_min_*):



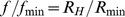
(9)Where *R_min_* depends on the cubic root of the molar mass (*M*) and non-hydrated volumes of the particle (*V*):



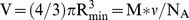
(10)


### NMR Experiments

NMR experiments were performed on a Bruker 600 MHz Avance III spectrometer equipped with a 5 mm triple resonance cryoprobe incorporating shielded z-axis gradient coils. All the heteronuclear ^13^C-^1^H correlation experiments were carried out at natural abundance. Pulsed field gradients were appropriately employed to achieve suppression of the solvent signal and spectral artifacts. The proton carrier was centered on the H_2_O frequency. Quadrature detection in the indirectly detected dimensions was obtained using the States-TPPI or the echo-antiecho method and the spectra were processed with the NMRPipe software [73] and analyzed using NMRView [74].

### Chemical shift assignment

In order to explore the conformational properties of E7 (1–40), all proton and carbon resonances of an unlabelled 3 mM sample of E7 (1–40) were assigned at 20°C, for which the best quality spectra were obtained, in the following solutions: H_2_O pHs 5.00 and 7.50, containing 5% D_2_O; TFE-d_2_ (50%), final pH 7.5; TFE-d_2_ (12%), final pH 5.0. All NMR samples contained 10 mM TCEP for avoiding oxidation in the course of the spectra acquisition. The experimental conditions were carefully controlled such that no oxidation or precipitation of the peptide occurred during measurements and that the pH of the sample remained stable.

Proton and carbon chemical shift assignments were achieved using a combination of homonuclear and heteronuclear standard 2D experiments. ^1^H-^1^H NOESY (100 and 250 ms mixing times), ^1^H-^1^H TOCSY (30 and 70 ms mixing times), ^1^H-^13^C HMQC, optimized to observe aliphatic or aromatic regions, and HMQC-TOCSY (70 ms mixing time) spectra were acquired. The NOESY experiments were acquired with 4096 (t2), and 1024 (t1) complex points, with spectral widths of 9615 and 6000 Hz in the direct and indirect dimensions, respectively. The TOCSY data sets consisted of 2048 (t2), and 800 (t1) complex points with the same spectral widths used in the NOESY experiments. The ^1^H-^13^C HMQC and ^1^H-^13^C HMQC-TOCSY data were collected with 2048 (t2), and 800 (t1) complex data points, and spectral widths of 9615 (^1^H), and 8906 (^13^C) Hz, respectively. The ^13^C carrier was centered at 40.0 ppm. For the aromatic 1H -13C HMQC, 2048 (t2), and 256 (t1) complex data points were collected, the spectral widths were of 9615 and 3774 Hz, respectively, and the carbon carrier was 130.0 ppm. Chemical shifts in water pH 5.0 were deposited in the Biological Magnetic Resonance Bank (BMRB code: 19269). For the other experimental conditions see Tables S1, S2 and S3.

### Transient secondary structures of E7 (1–40)

Typically, secondary chemical shifts are a convenient and accurate way to determine a polypeptide secondary structure [75]. However, E7 (1–40) exhibit shifts that are significantly smaller than in ordered proteins, as expected for an IDP. Therefore, to estimate the E7 (1–40) transient secondary structures, we decided to compare the chemical shifts of the domain observed in different experimental conditions that stabilize particular conformations of the peptide (diverse pHs and temperatures and presence of TFE as a co-solvent). Thus, we were able to investigate the secondary structure conformations that E7 (1–40) populates in solution. To assist in the study of the local E7 (1–40) conformational preferences, the ^1^J_CαHα_ coupling constants were also measured. In this case, proton coupled ^1^H -^13^C HMQC experiments were performed in H_2_O pH 7.5 (5% D_2_O) at 4°C; in H_2_O pH 5.0 (5% D_2_O) at 20°C; and in H_2_O TFE-d2 (50%) final pH 7.50 at 20°C. To increase resolution, the spectral width of the indirectly detected dimension was lowered to a fraction of the ^13^C spectral window. The alpha region was selected and the rest of the signals were folded into the reduced spectral window. Thus, the ^1^H-^13^C HMQCs were acquired with the ^13^C carbon carrier centered also at 40.0ppm, but with 2048 (t2), and 2048 (t1) complex data points, and spectral widths of 9615 (^1^H), and 2868 (^13^C) Hz, respectively.

### Diffusion measurements

The pulsed field gradient NMR self-diffusion measurements were performed using the PFG-SLED sequence [76]. Dioxane (10 µL, 2% in H_2_O) was added to the sample (300 µL) as internal standard [77]. The length of all pulses and delays in the sequence were held constant and 19 spectra were acquired with the strength of the diffusion gradient varying between 5% and 95% of its maximum value. The pulse gradient width was 4 ms, and the length of the diffusion delay was calibrated for the sample in order to give a maximal decay of 80–90% for the protein and dioxane signals (150 ms and 20 ms for E7N and dioxane respectively). The E7N intensity decrease was mostly homogeneous throughout the entire proton spectrum (except for the dioxane and TCEP signals). Therefore, we were able to fit the E7N (1–40) intensity decay by following both the aliphatic and the aromatic- amide regions of the spectrum. A T2 filter was used to selectively observe the dioxane signal, without interference of the protein, and therefore to reduce the experimental error, especially at high gradient strengths. The dioxane NMR spectra were acquired with 16 K complex points, and the protein NMR spectra with 4 K complex points. Hydrodynamic radii (R_H_) values for E7N in different experimental conditions were calculated as follows:



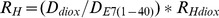
(11)Where D_diox_ and D_E7 (1–40)_ are the measured diffusion coefficients of dioxane and E7 (1–40), respectively, and R_Hdiox_ is the effective hydrodynamic radius of dioxane, taken to be 2.12 Å [77]. Theoretical hydrodynamic radii were calculated from the empirical equations for folded proteins:



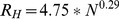
(12)


And for unfolded proteins:



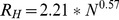
(13)Where N is the number of residues [51]. For IDPs, we used an equation that takes into account the main sequence determinants of the R_H_ [78]:



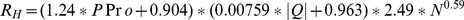
(14)Where PPro is the fraction of proline residues, 2/40 in E7 (1–40), and |Q| the absolute net charge. |Q| was calculated at pHs 5.0 and 7.5 using the Protein Calculator v3.3 software, which uses for the individual amino acids the pKa values for isolated residues.

## Supporting Information

Figure S1 **TFE titration curves followed by Far UV-CD at pH 7.5 and pH 5.0.** Rows represent the TFE titration curves for E7N and E7N sub-fragments. The name of the fragments is indicated at the right of each row. A) Far-UV CD spectra at different TFE percentage mixtures measured at pH 5.0. The arrows show the sense of change upon increasing TFE percentage. B) TFE Titration curves followed by ellipticity at 222 nm at different [TFE]/[buffer] molar ratios at pH 7.5 (black circles) and pH 5.0 (open circles). The data were fitted to a two-state coil-helix equilibrium model (see Materials and Methods and Table 2). For peptides E7 (21–29) and E7 (25–40), the data could not be reliably fitted to the two-state model due to the lack of a transition. The vertical dashed lines in the E7N panel show the molar ratio corresponding to 12%, 30% and 53% TFE, respectively. The buffers used for all spectra were 10 mM Tris.Cl buffer (pH 7.5) and 10 mM sodium formate buffer (pH 5.0). All measurements were performed at 20°C.
(TIF)

Figure S2 **pH induction of α-helix structure within the E7N domain.** Rows represent the pH transition curves at 30% TFE for E7N and the different sub-fragments. The name of the fragments is indicated at the right of each row. A) Far-UV CD spectra at different pH values after one hour of incubation. The arrows show the sense of change upon decreasing pH. B) pH equilibrium transition followed by molar ellipticity at 222 nm. The vertical dashed lines show the pH values selected for the CD and NMR studies. The buffers used for all spectra were 10 mM citrate phosphate with 30% TFE at different pH values ranging from 8.0 to 3.0. Fits for each signal obtained from global fitting of the data to Equation 4 are plotted as full lines. All measurements were performed at 20°C.
(TIF)

Figure S3
**Analytical ultracentrifugation of the E7N domain and CR1 and CR2 regions.** Sedimentation velocity profiles of E7 (1–40), E7 (1–20), and E7 (16–40) (left to right panels) at 50000 rpm and 20°C, in 50 mM Tris.Cl buffer at pH 7.5. A) Superposition of experimental (dots) and fitted (continuous line) profiles corrected for all systematic noise for E7 (1–40), E7 (1–20), and E7 (16–40) at 1.0, 3.4, and 2.2 mg ml^−1^ respectively. The last profile corresponds to 24 hours of sedimentation. The fit was obtained from the *c*(*s*) analysis of the SEDFIT program. For all peptides, the Lamm equation was simulated for 300 particles in the ranges (0 S, 10 S) and (0 S, 2 S), with a partial specific volume 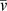 of 0.70, 0.72, and 0.68 ml g^−1^ for E7 (1–40), E7 (1–20), and E7 (16–40) respectively. Frictional ratio *f*/*f*_min_ was fitted for each sample. B) Corresponding c(s) distribution in the range 0–10 S. The signal was normalized to 1 cm optical path length. C) Superposition of the c(s) distributions for different concentrations of peptides in the range 0–2 S. The signal was normalized to 1 cm optical path length.(TIF)

Figure S4 **Analytical ultracentrifugation concentration dependency analysis.** Concentration dependency of *s* ^−1^ A) and *D*_app_ B). The linear regressions (lines) provide values, which are given in Table S1, for *s*_0_, and *D*_0app_. The dependence of the inverse of the sedimentation coefficient *s* ^−1^ with the concentration *c* (g ml^−1^) is analyzed by the equation *s* ^−1^ =  *s*_0_^−1^+*k*_s_ *s*_0_^−1^ *c*, where *s*_0_ is the sedimentation coefficient extrapolated to zero concentration, and *k*_s_ is the concentration dependence coefficient for *s* (ml g^−1^). For the apparent diffusion coefficient *D*_app_, the dependence with the concentration *c* (g ml^−1^) is analyzed by the equation *D*_app_  =  *D*_0app_+*k*_D_ *D*_0app_ *c*, where *D*_0app_ is the diffusion coefficient extrapolated to zero concentration, and *k*_D_ is the concentration dependence coefficient for *D*_app_ (ml g^−1^).
(TIF)

Figure S5 **Analysis of polyproline type II (pII) propensity within the E7N domain.** Difference spectrum between 5°C and 75°C for E7N and E7N sub-fragments performed in 10 mM Tris.Cl buffer at pH 7.5 (full line) and 10 mM sodium formate buffer at pH 5.0 (dotted line). The fragment names are indicated inside each panel.
(TIF)

Table S1 **^1^H and ^13^C chemical shifts assignments of the E7N in aqueous solution containing 10** **mM TCEP and 5% D_2_O at 20°C and pH 7.5.** ^a 1^H Chemical shifts are reported in ppm with an accuracy of ±0.02 ppm. ^13^C Chemical shifts are reported in ppm with an accuracy of ±0.1 ppm. ^b^Carbon chemical shifts first, and proton chemical shift in brackets. ^c^These signals may be interchangeable.
(DOC)

Table S2 **^1^H and ^13^C chemical shifts assignments of the E7N in aqueous solution containing 10** **mM TCEP, 12% TFE-** ***d*** **_2_ at 20°C and pH 5.0.** ^a 1^H Chemical shifts are reported in ppm with an accuracy of ±0.02 ppm. ^13^C Chemical shifts are reported in ppm with an accuracy of ±0.1 ppm. ^b^Carbon chemical shifts first, and proton chemical shift in brackets.
(DOC)

Table S3 **^1^H and ^13^C chemical shifts assignments of the E7N in a 1:1 H_2_O: TFE-** ***d*** **_2_ solution containing 5** **mM TCEP at 20°C and pH 7.5.** ^a 1^H Chemical shifts are reported in ppm with an accuracy of ±0.02 ppm. ^13^C Chemical shifts are reported in ppm with an accuracy of ±0.1 ppm. ^b^Carbon chemical shifts first, and proton chemical shift in brackets.
(DOC)

Table S4 **^1^J_CαHα_ scalar coupling constant values (in Hz) measured for E7N.** ^a^ The accuracy of the measurements is estimated to be ±0.5 Hz. ^b^TFE-*d_2_*. ^c^ n.d.: not determined.
(DOC)

## References

[1] Wright PE, Dyson HJ (1999) Intrinsically unstructured proteins: re-assessing the protein structure-function paradigm. J Mol Biol 293: 321–331. 10550212 10.1006/jmbi.1999.3110

[2] Uversky VN (2012) Unusual biophysics of intrinsically disordered proteins. Biochim Biophys Acta 1834: 932–951. 23269364 10.1016/j.bbapap.2012.12.008

[3] Uversky VN (2002) What does it mean to be natively unfolded? Eur J Biochem 269: 2–12. 11784292 10.1046/j.0014-2956.2001.02649.x

[4] Tompa P (2002) Intrinsically unstructured proteins. Trends Biochem Sci 27: 527–533. 12368089 10.1016/s0968-0004(02)02169-2

[5] Kriwacki RW, Hengst L, Tennant L, Reed SI, Wright PE (1996) Structural studies of p21Waf1/Cip1/Sdi1 in the free and Cdk2-bound state: conformational disorder mediates binding diversity. Proc Natl Acad Sci U S A 93: 11504–11509. 8876165 10.1073/pnas.93.21.11504 PMC38087

[6] Uversky VN, Gillespie JR, Millett IS, Khodyakova AV, Vasiliev AM, et al (1999) Natively unfolded human prothymosin alpha adopts partially folded collapsed conformation at acidic pH. Biochemistry 38: 15009–15016. 10555983 10.1021/bi990752+

[7] Xue B, Dunker AK, Uversky VN (2012) Orderly order in protein intrinsic disorder distribution: disorder in 3500 proteomes from viruses and the three domains of life. J Biomol Struct Dyn 30: 137–149. 22702725 10.1080/07391102.2012.675145

[8] Uversky VN, Oldfield CJ, Dunker AK (2008) Intrinsically disordered proteins in human diseases: introducing the D2 concept. Annu Rev Biophys 37: 215–246. 18573080 10.1146/annurev.biophys.37.032807.125924

[9] Uversky VN, Gillespie JR, Fink AL (2000) Why are “natively unfolded” proteins unstructured under physiologic conditions? Proteins 41: 415–427. 11025552 10.1002/1097-0134(20001115)41:3<415::aid-prot130>3.0.co;2-7

[10] Dosztanyi Z, Csizmok V, Tompa P, Simon I (2005) IUPred: web server for the prediction of intrinsically unstructured regions of proteins based on estimated energy content. Bioinformatics 21: 3433–3434. 15955779 10.1093/bioinformatics/bti541

[11] Uversky VN, Dunker AK (2012) Intrinsically Disordered Protein Analysis: Methods and experimental tools. Methods in Molecular Biology 895. Springer Protocols: Humana Press. 511p.

[12] Davey NE, Van Roey K, Weatheritt RJ, Toedt G, Uyar B, et al (2012) Attributes of short linear motifs. Mol Biosyst 8: 268–281. 21909575 10.1039/c1mb05231d

[13] Meszaros B, Dosztanyi Z, Simon I (2012) Disordered binding regions and linear motifs – bridging the gap between two models of molecular recognition. PLoS One 7 (10): e46829. 10.1371/journal.pone.0046829 PMC3463566 23056474

[14] Fuxreiter M, Simon I, Friedrich P, Tompa P (2004) Preformed structural elements feature in partner recognition by intrinsically unstructured proteins. J Mol Biol 338: 1015–1026. 15111064 10.1016/j.jmb.2004.03.017

[15] Tokuriki N, Oldfield CJ, Uversky VN, Berezovsky IN, Tawfik DS (2009) Do viral proteins possess unique biophysical features? Trends Biochem Sci 34: 53–59. 19062293 10.1016/j.tibs.2008.10.009

[16] Uversky VN, Longhi S (2012) Flexible Viruses: Structural Disorder in Viral Proteins. Wiley Series in Protein Peptide Science. 494p.

[17] Howley PM (1996) *Papillomavirinae*: The viruses and Their Replication. In Fields BN, Knipe DM, Howlwy PM, editors. Fundamental Virology: Philadelphia: Lippincott-Raven Publishers. pp. 947–978.

[18] zur Hausen H (1996) Papillomavirus infections – a major cause of human cancers. Biochim Biophys Acta 1288: F55–78. 8876633 10.1016/0304-419x(96)00020-0

[19] Stubenrauch F, Laimins LA (1999) Human papillomavirus life cycle: active and latent phases. Semin Cancer Biol 9: 379–386. 10712884 10.1006/scbi.1999.0141

[20] Munger K, Howley PM (2002) Human papillomavirus immortalization and transformation functions. Virus Res 89: 213–228. 12445661 10.1016/s0168-1702(02)00190-9

[21] Moody CA, Laimins LA (2010) Human papillomavirus oncoproteins: pathways to transformation. Nat Rev Cancer 10: 550–560. 20592731 10.1038/nrc2886

[22] Munger K, Phelps WC (1993) The human papillomavirus E7 protein as a transforming and transactivating factor. Biochim Biophys Acta 1155: 111–123. 8389201 10.1016/0304-419x(93)90025-8

[23] McLaughlin-Drubin ME, Munger K (2009) The human papillomavirus E7 oncoprotein. Virology 384: 335–344. 19007963 10.1016/j.virol.2008.10.006 PMC2661820

[24] Chemes LB, Glavina J, Faivovich J, de Prat-Gay G, Sanchez IE (2012) Evolution of linear motifs within the papillomavirus E7 oncoprotein. J Mol Biol 422: 336–346. 22683353 10.1016/j.jmb.2012.05.036

[25] Dantur K, Alonso L, Castano E, Morelli L, Centeno-Crowley JM, et al (2009) Cytosolic accumulation of HPV16 E7 oligomers supports different transformation routes for the prototypic viral oncoprotein: the amyloid-cancer connection. Int J Cancer 125: 1902–1911. 19598264 10.1002/ijc.24579

[26] Alonso LG, Garcia-Alai MM, Nadra AD, Lapena AN, Almeida FL, et al (2002) High-risk (HPV16) human papillomavirus E7 oncoprotein is highly stable and extended, with conformational transitions that could explain its multiple cellular binding partners. Biochemistry 41: 10510–10518. 12173938 10.1021/bi025579n

[27] Garcia-Alai MM, Alonso LG, de Prat-Gay G (2007) The N-terminal module of HPV16 E7 is an intrinsically disordered domain that confers conformational and recognition plasticity to the oncoprotein. Biochemistry 46: 10405–10412. 17715947 10.1021/bi7007917

[28] Liu X, Clements A, Zhao K, Marmorstein R (2006) Structure of the human Papillomavirus E7 oncoprotein and its mechanism for inactivation of the retinoblastoma tumor suppressor. J Biol Chem 281: 578–586. 16249186 10.1074/jbc.M508455200

[29] Fassolari M, Chemes LB, Gallo M, Smal C, Sanchez IE, et al (2013) Minute-timescale prolyl isomerization governs antibody recognition of an intrinsically disordered immunodominant epitope. J Biol Chem 288: 13110–13123. 23504368 10.1074/jbc.M112.444554 PMC3642352

[30] Uversky VN, Roman A, Oldfield CJ, Dunker AK (2006) Protein intrinsic disorder and human papillomaviruses: increased amount of disorder in E6 and E7 oncoproteins from high risk HPVs. J Proteome Res 5: 1829–1842. 16889404 10.1021/pr0602388

[31] Barbosa MS, Lowy DR, Schiller JT (1989) Papillomavirus polypeptides E6 and E7 are zinc-binding proteins. J Virol 63: 1404–1407. 2536841 10.1128/jvi.63.3.1404-1407.1989 PMC247840

[32] Dyson N, Guida P, Munger K, Harlow E (1992) Homologous sequences in adenovirus E1A and human papillomavirus E7 proteins mediate interaction with the same set of cellular proteins. J Virol 66: 6893–6902. 1331501 10.1128/jvi.66.12.6893-6902.1992 PMC240306

[33] Chellappan S, Kraus VB, Kroger B, Munger K, Howley PM, et al (1992) Adenovirus E1A, simian virus 40 tumor antigen, and human papillomavirus E7 protein share the capacity to disrupt the interaction between transcription factor E2F and the retinoblastoma gene product. Proc Natl Acad Sci U S A 89: 4549–4553. 1316611 10.1073/pnas.89.10.4549 PMC49120

[34] Davey NE, Trave G, Gibson TJ (2011) How viruses hijack cell regulation. Trends Biochem Sci 36: 159–169. 21146412 10.1016/j.tibs.2010.10.002

[35] Dick FA (2007) Structure-function analysis of the retinoblastoma tumor suppressor protein – is the whole a sum of its parts? Cell Div 2: 26. 17854503 10.1186/1747-1028-2-26 PMC2082274

[36] Dyer MD, Murali TM, Sobral BW (2008) The landscape of human proteins interacting with viruses and other pathogens. PLoS Pathog 4: e32. 18282095 10.1371/journal.ppat.0040032 PMC2242834

[37] Chemes LB, Glavina J, Alonso LG, Marino-Buslje C, de Prat-Gay G, et al (2012) Sequence evolution of the intrinsically disordered and globular domains of a model viral oncoprotein. PLoS One 7: e47661. 23118886 10.1371/journal.pone.0047661 PMC3485249

[38] Garcia-Alai MM, Gallo M, Salame M, Wetzler DE, McBride AA, et al (2006) Molecular basis for phosphorylation-dependent, PEST-mediated protein turnover. Structure 14: 309–319. 16472750 10.1016/j.str.2005.11.012

[39] Alonso LG, Garcia-Alai MM, Smal C, Centeno JM, Iacono R, et al (2004) The HPV16 E7 viral oncoprotein self-assembles into defined spherical oligomers. Biochemistry 43: 3310–3317. 15035602 10.1021/bi036037o

[40] Smal C, Alonso LG, Wetzler DE, Heer A, de Prat Gay G (2012) Ordered self-assembly mechanism of a spherical oncoprotein oligomer triggered by zinc removal and stabilized by an intrinsically disordered domain. PLoS One 7: e36457. 22590549 10.1371/journal.pone.0036457 PMC3348928

[41] Alonso LG, Smal C, Garcia-Alai MM, Chemes L, Salame M, et al (2006) Chaperone holdase activity of human papillomavirus E7 oncoprotein. Biochemistry 45: 657–667. 16411741 10.1021/bi0522549

[42] Smal C, Wetzler DE, Dantur KI, Chemes LB, Garcia-Alai MM, et al (2009) The human papillomavirus E7-E2 interaction mechanism in vitro reveals a finely tuned system for modulating available E7 and E2 proteins. Biochemistry 48: 11939–11949. 19899811 10.1021/bi901415k

[43] Chemes LB, Sanchez IE, Alonso LG, de Prat-Gay G (2012) Intrinsic Disorder in the Human Papillomavirus E7 Protein. In: Longhi S, Uversky VN, editors. Flexible Viruses: Structural Disorder in Viral Proteins: Wiley Series in Protein and Peptide Science. pp. 313–346.

[44] Lee JO, Russo AA, Pavletich NP (1998) Structure of the retinoblastoma tumour-suppressor pocket domain bound to a peptide from HPV E7. Nature 391: 859–865. 9495340 10.1038/36038

[45] Singh M, Krajewski M, Mikolajka A, Holak TA (2005) Molecular determinants for the complex formation between the retinoblastoma protein and LXCXE sequences. J Biol Chem 280: 37868–37876. 16118215 10.1074/jbc.M504877200

[46] Chen K, Liu Z, Kallenbach NR (2004) The polyproline II conformation in short alanine peptides is noncooperative. Proc Natl Acad Sci U S A 101: 15352–15357. 15489268 10.1073/pnas.0406657101 PMC524463

[47] Jasanoff A, Fersht AR (1994) Quantitative determination of helical propensities from trifluoroethanol titration curves. Biochemistry 33: 2129–2135. 8117669 10.1021/bi00174a020

[48] Pace CN, Grimsley GR, Scholtz JM (2009) Protein ionizable groups: pK values and their contribution to protein stability and solubility. J Biol Chem 284: 13285–13289. 19164280 10.1074/jbc.R800080200 PMC2679426

[49] Ma K, Clancy EL, Zhang Y, Ray DG, Wollenberg K, et al (1999) Residue-Specific pKa Measurements of the â-Peptide and Mechanism of pH-Induced Amyloid Formation. J Am Chem Soc 121: 8698–8706.

[50] Salvay AG, Communie G, Ebel C (2012) Sedimentation velocity analytical ultracentrifugation for intrinsically disordered proteins. In: Intrinsically Disordered Protein Analysis: Methods in Molecular Biology Uversky VN, Dunker AK, editors. 896: pp. 91–105. 10.1007/978-1-4614-3704-8_6 22821519

[51] Wilkins DK, Grimshaw SB, Receveur V, Dobson CM, Jones JA, et al (1999) Hydrodynamic radii of native and denatured proteins measured by pulse field gradient NMR techniques. Biochemistry 38: 16424–16431. 10600103 10.1021/bi991765q

[52] Shen Y, Bax A (2010) Prediction of Xaa-Pro peptide bond conformation from sequence and chemical shifts. J Biomol NMR 46: 199–204. 20041279 10.1007/s10858-009-9395-y PMC2847849

[53] Kashtanov S, Borcherds W, Wu H, Daughrill GW, Ytreberg FM (2012) Using chemical shifts to assess transient secondary structure and generate ensemble structure of intrinsically disordered proteins. In: Intrinsically Disordered Protein Analysis: Methods in Molecular Biology Uversky VN, Dunker K, editors. 895: pp. 139–152. 10.1007/978-1-61779-927-3_11 22760318

[54] Eliezer D (2009) Biophysical characterization of intrinsically disordered proteins. Curr Opin Struct Biol 19: 23–30. 19162471 10.1016/j.sbi.2008.12.004 PMC2728036

[55] Chen YH, Yang JT, Chau KH (1974) Determination of the helix and beta form of proteins in aqueous solution by circular dichroism. Biochemistry 13: 3350–3359. 4366945 10.1021/bi00713a027

[56] Shi Z, Olson CA, Rose GD, Baldwin RL, Kallenbach NR (2002) Polyproline II structure in a sequence of seven alanine residues. Proc Natl Acad Sci U S A 99: 9190–9195. 12091708 10.1073/pnas.112193999 PMC123116

[57] Schmidt JM, Zhou S, Rowe ML, Howard MJ, Williamson RA, et al (2011) One-bond and two-bond J couplings help annotate protein secondary-structure motifs: J-coupling indexing applied to human endoplasmic reticulum protein ERp18. Proteins 79: 428–443. 21117079 10.1002/prot.22893

[58] Shi Z, Woody RW, Kallenbach NR (2002) Is polyproline II a major backbone conformation in unfolded proteins? Adv Protein Chem 62: 163–240. 12418104 10.1016/s0065-3233(02)62008-x

[59] Makowska J, Rodziewicz-Motowidlo S, Baginska K, Makowski M, Vila JA (2007) et.al (2007) Further Evidence for the Absence of Polyproline II Stretch in the XAO Peptide. Biophys J 92: 2904–2917. 17277185 10.1529/biophysj.106.097550 PMC1831701

[60] Lam SL, Hsu VL (2003) NMR identification of left-handed polyproline type II helices. Biopolymers 69: 270–281. 12767128 10.1002/bip.10354

[61] Vuister GW, Delaglio F, Bax A (1993) The use of 1JCαHα coupling constants as a probe for protein backbone conformation. J Biol NMR 3: 67–80. 10.1007/BF00242476 8448436

[62] Chemes LB, Alonso LG, Noval MG, de Prat-Gay G (2012) Circular dichroism techniques for the analysis of intrinsically disordered proteins and domains. In: Intrinsically Disordered Protein Analysis: Methods in Molecular Biology Uversky VN, Dunker K, editors. 895: pp. 387–404. 10.1007/978-1-61779-927-3_22 22760329

[63] Mikhonin AV, Myshakina NS, Bykov SV, Asher SA (2005) UV resonance Raman determination of polyproline II, extended 2.5(1)-helix, and beta-sheet Psi angle energy landscape in poly-L-lysine and poly-L-glutamic acid. J Am Chem Soc 127: 7712–7720. 15913361 10.1021/ja044636s

[64] Kim MK, Kang YK (1999) Positional preference of proline in alpha-helices. Protein Sci 8: 1492–1499. 10422838 10.1110/ps.8.7.1492 PMC2144370

[65] Liu X, Marmorstein R (2007) Structure of the retinoblastoma protein bound to adenovirus E1A reveals the molecular basis for viral oncoprotein inactivation of a tumor suppressor. Genes Dev 21: 2711–2716. 17974914 10.1101/gad.1590607 PMC2045126

[66] Lee C, Chang JH, Lee HS, Cho Y (2002) Structural basis for the recognition of the E2F transactivation domain by the retinoblastoma tumor suppressor. Genes Dev 16: 3199–3212. 12502741 10.1101/gad.1046102 PMC187509

[67] Kim HY, Ahn BY, Cho Y (2001) Structural basis for the inactivation of retinoblastoma tumor suppressor by SV40 large T antigen. Embo J 20: 295–304. 11226179 10.1093/emboj/20.1.295 PMC140208

[68] Uversky VN (2011) Intrinsically disordered proteins may escape unwanted interactions via functional misfolding. Biochim Biophys Acta 1814: 693–712. 21440685 10.1016/j.bbapap.2011.03.010

[69] Chemes LB, Sanchez IE, de Prat-Gay G (2011) Kinetic recognition of the retinoblastoma tumor suppressor by a specific protein target. J Mol Biol 412: 267–284. 21787785 10.1016/j.jmb.2011.07.015

[70] Chemes LB, Sanchez IE, Smal C, de Prat-Gay G (2010) Targeting mechanism of the retinoblastoma tumor suppressor by a prototypical viral oncoprotein. Structural modularity, intrinsic disorder and phosphorylation of human papillomavirus E7. Febs J 277: 973–988. 20088881 10.1111/j.1742-4658.2009.07540.x

[71] Nicolau-Junior N, Giuliatti S (2013) Modeling and molecular dynamics of the intrinsically disordered e7 proteins from high- and low-risk types of human papillomavirus. Journal of Molecular Modeling In Press. 10.1007/s00894-013-1915-8 23864166

[72] Thurlkill RL, Grimsley GR, Scholtz JM, Pace CN (2006) pK values of the ionizable groups of proteins. Protein Sci 15: 1214–1218. 16597822 10.1110/ps.051840806 PMC2242523

[73] Delaglio F, Grzesiek S, Vuister GW, Zhu G, Pfeifer J, et al (1995) NMRPipe: a multidimensional spectral processing system based on UNIX pipes. J Biomol NMR 6: 277–293. 8520220 10.1007/BF00197809

[74] Johnson BA (2004) Using NMRView to visualize and analyze the NMR spectra of macromolecules. In: Protein NMR Techniques: Methods Mol Biol Downing AK, editor. 278: pp. 313–352. 10.1385/1-59259-809-9:313 15318002

[75] Whishart DS, Sykes BD (1994) Chemical shifts as a tool for structure determination. In: Nuclear Magnetic Resonance: Methods Enzymol James TL, Oppenheimer NJ, editors. 293: pp. 363–392. 10.1016/s0076-6879(94)39014-2 7830591

[76] Merrill MR (1993) NMR diffusion measurements using a composite gradient PGSE sequence. J Magn Reson 103: 223–225.

[77] Jones JA, Wilkins DK, Smith LJ, Dobson CM (1997) Characterization of protein unfolding by NMR diffusion measurements. Journal of Biomolecular NMR 10: 199–203.

[78] Marsh JA, Forman-Kay JD (2010) Sequence determinants of compaction in intrinsically disordered proteins. Biophys J 98: 2383–2390. 20483348 10.1016/j.bpj.2010.02.006 PMC2872267

