# Supplementary figures and images for: Conformational Dissection of a Viral Intrinsically Disordered Domain Involved in Cellular Transformation
Source: PLoS One. 2013 Sep 27;8(9):e72760. doi: 10.1371/journal.pone.0072760 (PMC3785498; doi:10.1371/journal.pone.0072760)

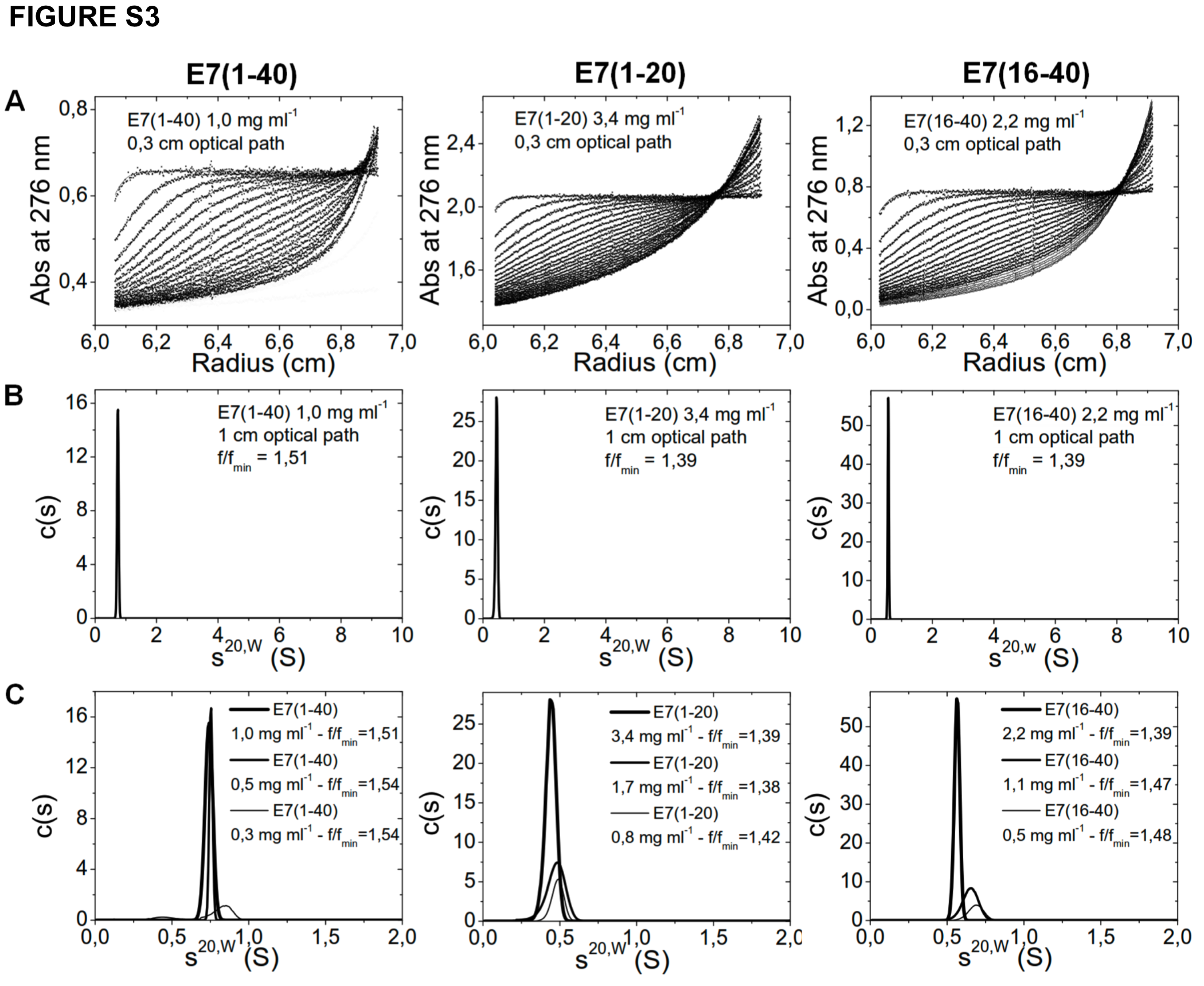

Supplement: Figure S2 — pH induction of α-helix structure within the E7N domain. Rows represent the pH transition curves at 30% TFE for E7N and the different sub-fragments. The name of the fragments is indicated at the right of each row. A) Far-UV CD spectra at different pH values after one hour of incubation. The arrows show the sense of change upon decreasing pH. B) pH equilibrium transition followed by molar ellipticity at 222 nm. The vertical dashed lines show the pH values selected for the CD and NMR studies. The buffers used for all spectra were 10 mM citrate phosphate with 30% TFE at different pH values ranging from 8.0 to 3.0. Fits for each signal obtained from global fitting of the data to Equation 4 are plotted as full lines. All measurements were performed at 20°C. (TIF) [file pone.0072760.s002.tif]

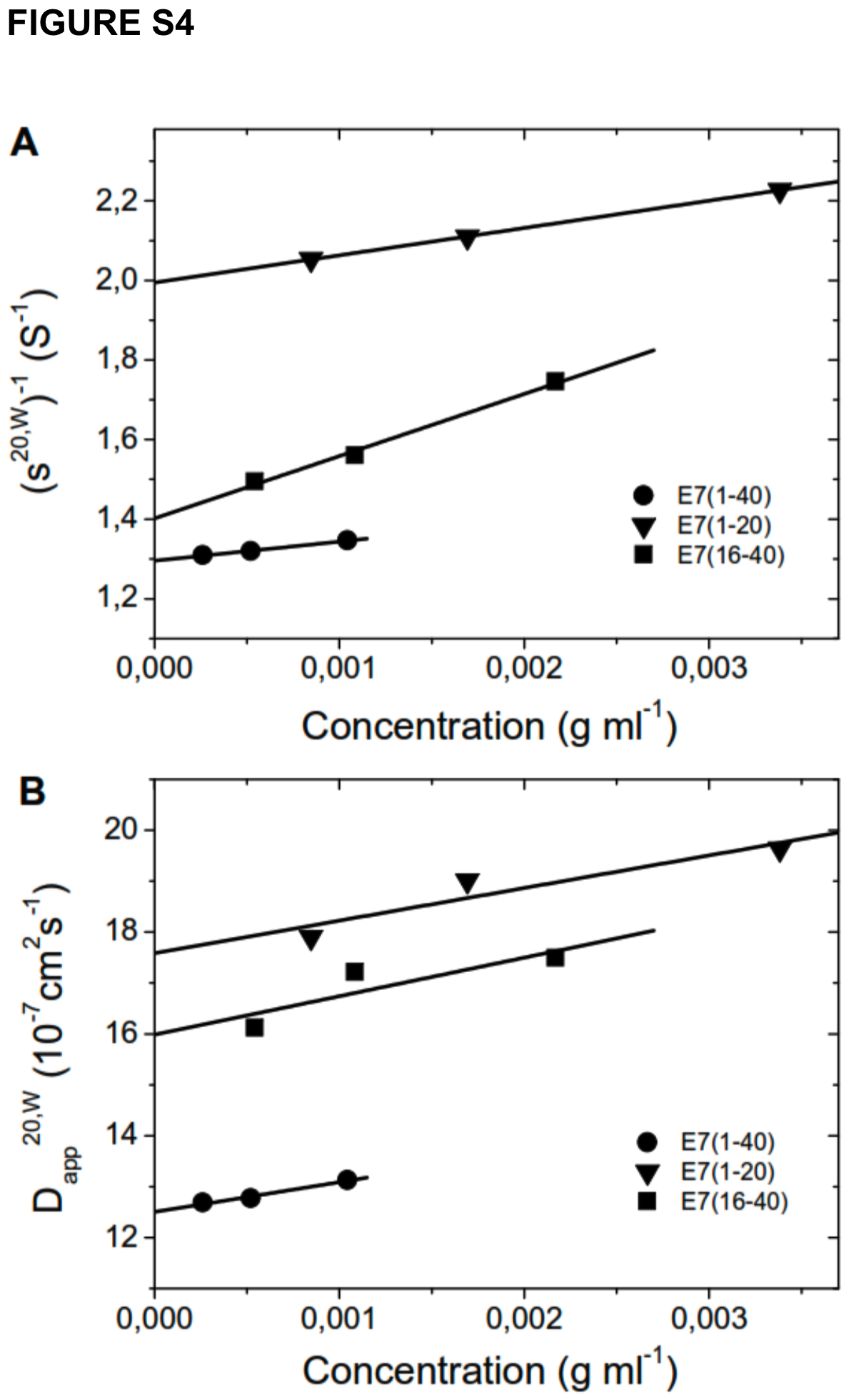

Supplement: Figure S4 — Analytical ultracentrifugation concentration dependency analysis. Concentration dependency of s −1 A) and D app B). The linear regressions (lines) provide values, which are given in Table S1, for s 0, and D 0app. The dependence of the inverse of the sedimentation coefficient s −1 with the concentration c (g ml−1) is analyzed by the equation s −1 = s 0 −1+k s s 0 −1 c, where s 0 is the sedimentation coefficient extrapolated to zero concentration, and k s is the concentration dependence coefficient for s (ml g−1). For the apparent diffusion coefficient D app, the dependence with the concentration c (g ml−1) is analyzed by the equation D app = D 0app+k D D 0app c, where D 0app is the diffusion coefficient extrapolated to zero concentration, and k D is the concentration dependence coefficient for D app (ml g−1). (TIF) [file pone.0072760.s004.tif]
