# Supplementary material for: Conformational Dissection of a Viral Intrinsically Disordered Domain Involved in Cellular Transformation
Source: PLoS One. 2013 Sep 27;8(9):e72760. doi: 10.1371/journal.pone.0072760 (PMC3785498; doi:10.1371/journal.pone.0072760)
Supplement: Table S1 — 1H and 13C chemical shifts assignments of the E7N in aqueous solution containing 10 mM TCEP and 5% D2O at 20°C and pH 7.5. a 1H Chemical shifts are reported in ppm with an accuracy of ±0.02 ppm. 13C Chemical shifts are reported in ppm with an accuracy of ±0.1 ppm. bCarbon chemical shifts first, and proton chemical shift in brackets. cThese signals may be interchangeable. (DOC) [file pone.0072760.s006.doc]

Table S1.

| Residue | NH | ^13^C_α_ | ^1^H_α_ | ^13^C_β_ | ^1^H_β_ | Others^b^ |
| --- | --- | --- | --- | --- | --- | --- |
| **M1** |  | 54.9 | 3.88 | 33.3 | 1.99 | γ 30.6 (2.49/2.49), ε 16.7 (2.05) |
| **H2** |  | 56.4 | 4.57 | 30.1 | 3.11/3.08 |  |
| **G3** |  | 44.3 | 3.96/3.83 |  |  |  |
| **D4** | 8.26 | 53.9 | 4.62 | 40.7 | 2.64/2.64 |  |
| **T5** | 8.16 | 59.3 | 4.57 | 69.2 | 4.12 | δ_2_ 20.8 (1.20) |
| **P6** |  | 62.8 | 4.44 | 31.6 | 2.26/1.87 | γ 26.8 (1.97/1.97), δ 50.5 (3.81/3.68) |
| **T7** | 8.26 | 61.5 | 4.23 | 69.3 | 4.11 | δ_2_ 21.1 (1.13) |
| **L8** | 8.23 | 54.7 | 4.29 | 41.7 | 1.49/1.54 | γ 26.4 (1.50), δ_1_ 23.0 (0.80), δ_2_ 24.2 (0.86) |
| **H9** | 8.21 | 55.7 | 4.52 | 30.2 | 3.00/2.95 |  |
| **E10** | 8.35 | 56.5 | 4.13 | 29.6 | 1.89/1.85 | γ 35.7 (2.09/2.09) |
| **Y11** | 8.15 | 57.4 | 4.53 | 38.0 | 3.03/2.94 |  |
| **M12** | 8.15 | 54.9 | 4.37 | 32.2 | 2.02/1.92 | γ 31.4 (2.46/2.41), ε 16.3 (2.03) |
| **L13** | 8.02 | 54.8 | 4.24 | 41.9 | 1.58/1.54 | γ 26.4 (1.57), δ_1_ 22.9 (0.83), δ_2_ 24.3 (0.89) |
| **D14** | 8.24 | 53.7 | 4.55 | 40.4 | 2.66/2.74 |  |
| **L15** | 8.00 | 54.4 | 4.30 | 41.9 | 1.58/1.54 | γ 26.3 (1.55), δ_1_ 22.9 (0.82), δ_2_ 24.5 (0.88) |
| **Q16** | 8.39 | 53.0 | 4.57 | 28.3 | 2.08/1.91 | γ 32.9 (2.31/2.31) |
| **P17** |  | 62.6 | 4.39 | 31.5 | 2.26/1.89 | γ 26.8 (1.97/1.97), δ 50.0 (3.71/3.63) |
| **E18** | 8.63 | 56.2 | 4.29 | 29.7 | 2.04/1.94 | γ 35.8 (2.29/2.25) |
| **T19** | 8.25 | 61.1 | 4.40 | 69.3 | 4.23 | δ_2_ 21.0 (1.17) |
| **T20** | 8.18 | 61.4 | 4.31 | 69.3 | 4.19 | δ_2_ 20.9 (1.16) |
| **D21** | 8.16 | 53.9 | 4.56 | 40.4 | 2.64/2.56 |  |
| **L22** | 8.07 | 54.8 | 4.21 | 41.8 | 1.45/1.38 | γ 26.3 (1.47), δ_1_ 22.9 (0.77), δ_2_ 24.3 (0.84) |
| **Y23** | 8.15 | 57.4 | 4.46 | 38.0 | 2.92/2.87 |  |
| **C24** | 7.99 | 57.7 | 4.38 | 27.3 | 2.75/2.75 |  |
| **Y25** | 8.16 | 57.7 | 4.48 | 38.1 | 3.04/2.91 |  |
| **E26** | 8.26 | 56.0 | 4.19 | 29.9 | 1.95/1.86 | γ 35.8 (2.18/2.18) |
| **Q27** | 8.29 | 55.1 | 4.26 | 28.9 | 2.05/1.95 | γ 33.8 (2.31/2.31) |
| **L28** | 8.31 | 54.5 | 4.32 | 41.8 | 1.61/1.54 | γ 26.4 (1.58), δ_1_ 22.8 (0.82), δ_2_ 24.4 (0.88) |
| **N29** | 8.45 | 52.6 | 4.71 | 38.7 | 2.79/2.69 |  |
| **D30** | 8.37 | 53.7 | 4.61 | 40.7 | 2.69/2.65 |  |
| **S31** | 8.32 | 57.7 | 4.48 | 63.3 | 3.89/3.85 |  |
| **S32** | 8.46 | 58.0 | 4.45 | 63.4 | 3.87/3.85 |  |
| **E33** | 8.47 | 56.0 | 4.30 | 29.8 | 2.05/1.89 | γ 35.7 (2.24/2.24) |
| **E34** | 8.39 | 55.9 | 4.26 | 29.7 | 2.00/1.88 | γ 35.7 (2.24/2.24) |
| **E35** | 8.44 | 55.9 | 4.27 | 30.1 | 1.89/2.02 | γ 35.7 (2.25/2.22) |
| **D36** | 8.41 | 53.9 | 4.55 | 40.9 | 2.64/2.56 |  |
| **E37** | 8.39 | 55.9 | 4.26 | 29.8 | 2.00/1.89 | γ 35.7 (2.25/2.20) |
| **I38** | 8.25 | 60.8 | 4.22 | 38.2 | 1.83 | γ_1_ 26.8 (1.42/1.16), δ_1_ 12.3 (0.83), γ_2_ 16.8 (0.86) |
| **D39** | 8.53 | 54.1 | 4.53 | 40.4 | 2.71/2.64 |  |
| **G40** | 8.39 | 44.7 | 3.87/3.87 |  |  |  |
