# Supplementary material for: Conformational Dissection of a Viral Intrinsically Disordered Domain Involved in Cellular Transformation
Source: PLoS One. 2013 Sep 27;8(9):e72760. doi: 10.1371/journal.pone.0072760 (PMC3785498; doi:10.1371/journal.pone.0072760)
Supplement: Table S2 — 1H and 13C chemical shifts assignments of the E7N in aqueous solution containing 10 mM TCEP, 12% TFE- d 2 at 20°C and pH 5.0. a 1H Chemical shifts are reported in ppm with an accuracy of ±0.02 ppm. 13C Chemical shifts are reported in ppm with an accuracy of ±0.1 ppm. bCarbon chemical shifts first, and proton chemical shift in brackets. (DOC) [file pone.0072760.s007.doc]

**Table S2.**

| Residue | NH | ^13^C_α_ | ^1^H_α_ | ^13^C_β_ | ^1^H_β_ | Others^b^ |
| --- | --- | --- | --- | --- | --- | --- |
| **M1** |  | 54.5 | 4.11 | 32.3 | 2.09 | γ 30.3 (2.52/2.52), ε 16.2 (2.03) |
| **H2** |  | 55.6 | 4.57 | 28.3 | 3.21/3.21 |  |
| **G3** |  | 44.5 | 3.90/3.83 |  |  |  |
| **D4** |  | 53.7 | 4.58 | 40.6 | 2.65/2.65 |  |
| **T5** |  | 59.3 | 4.53 | 69.2 | 4.14 | δ_2_ 20.8 (1.18) |
| **P6** |  | 63.0 | 4.38 | 31.5 | 2.24/1.85 | γ 26.8 (1.95/1.95), δ 50.5 (3.79/3.69) |
| **T7** |  | 61.4 | 4.20 | 69.4 | 4.10 | δ_2_ 21.1 (1.15) |
| **L8** |  | 55.2 | 4.20 | 41.7 | 1.58/1.52 | γ 26.4 (1.49), δ_1_ 23.2 (0.80), δ_2_ 24.1 (0.86) |
| **H9** |  | 55.3 | 4.48 | 28.4 | 3.11/3.08 |  |
| **E10** |  | 56.8 | 4.05 | 29.1 | 1.90/1.86 | γ 35.1 (2.16/2.16) |
| **Y11** |  | 57.9 | 4.41 | 37.9 | 3.05/2.93 |  |
| **M12** |  | 55.3 | 4.26 | 32.0 | 2.02/1.95 | γ 31.5 (2.48/2.43), ε 16.4 (2.02) |
| **L13** |  | 55.1 | 4.15 | 41.7 | 1.58/1.52 | γ 26.5 (1.54), δ_1_ 29.9 (0.80), δ_2_ 24.3 (0.86) |
| **D14** |  | 53.5 | 4.50 | 39.8 | 2.72/2.59 |  |
| **L15** |  | 54.5 | 4.22 | 41.9 | 1.57/1.53 | γ 26.4 (1.55), δ_1_ 22.8 (0.79), δ_2_ 24.5 (0.84) |
| **Q16** |  | 53.0 | 4.51 | 28.3 | 2.091.91 | γ 33.1 (2.32/2.32) |
| **P17** |  | 62.9 | 4.32 | 31.5 | 2.23/1.87 | γ 26.9 (1.95/1.95), δ 50.1 (3.68/3.61) |
| **E18** |  | 56.2 | 4.24 | 29.1 | 2.05/1.94 | γ 34.7 (2.32/2.32) |
| **T19** |  | 61.3 | 4.32 | 69.1 | 4.20 | δ_2_ 21.0 (1.15) |
| **T20** |  | 61.8 | 4.22 | 69.2 | 4.24 | δ_2_ 20.9 (1.14) |
| **D21** |  | 53.8 | 4.58 | 39.9 | 2.70/2.62 |  |
| **L22** |  | 55.2 | 4.13 | 41.7 | 1.44/1.36 | γ 26.3 (1.46), δ_1_ 23.0 (0.75), δ_2_ 24.3 (0.81) |
| **Y23** |  | 57.9 | 4.39 | 37.9 | 2.96/2.88 |  |
| **C24** |  | 58.4 | 4.28 | 27.1 | 2.77/2.77 |  |
| **Y25** |  | 58.1 | 4.40 | 38.0 | 2.95/2.95 |  |
| **E26** |  | 56.3 | 4.11 | 29.1 | 1.96/1.87 | γ 34.4 (2.25/2.25) |
| **Q27** |  | 55.5 | 4.16 | 28.7 | 2.04/1.93 | γ 33.4 (2.26/2.26) |
| **L28** |  | 54.8 | 4.22 | 41.8 | 1.57/1.52 | γ 26.4 (1.53), δ_1_ 22.8 (0.79), δ_2_ 24.2(0.86) |
| **N29** |  | 52.7 | 4.61 | 38.6 | 2.75/2.65 |  |
| **D30** |  | 53.5 | 4.58 | 40.1 | 2.69/2.69 |  |
| **S31** |  | 58.2 | 4.36 | 63.2 | 3.91/3.86 |  |
| **S32** |  | 58.4 | 4.35 | 63.2 | 3.88/3.88 |  |
| **E33** |  | 56.0 | 4.24 | 29.0 | 2.05/1.91 | γ 34.2 (2.31/2.31) |
| **E34** |  | 56.0 | 4.20 | 29.9 | 2.03/1.91 | γ 34.0 (2.32/2.32) |
| **E35** |  | 56.0 | 4.20 | 29.0 | 2.04/1.92 | γ 34.1 (2.33/2.33) |
| **D36** |  | 53.8 | 4.51 | 39.8 | 2.72/2.65 |  |
| **E37** |  | 55.8 | 4.22 | 28.9 | 3.22/3.22 | γ 34.1 (2.32/2.32) |
| **I38** |  | 60.9 | 4.03 | 38.2 | 1.84 | γ_1_ 26.8 (1.43/1.15), δ_1_ 11.9 (0.80), γ_2_ 16.8 (0.84) |
| **D39** |  | 53.7 | 4.58 | 40.0 | 2.64/2.64 |  |
| **G40** |  | 44.6 | 3.84 | 3.80/3.80 |  |  |
