# Supplementary material for: Conformational Dissection of a Viral Intrinsically Disordered Domain Involved in Cellular Transformation
Source: PLoS One. 2013 Sep 27;8(9):e72760. doi: 10.1371/journal.pone.0072760 (PMC3785498; doi:10.1371/journal.pone.0072760)
Supplement: Table S3 — 1H and 13C chemical shifts assignments of the E7N in a 1:1 H2O: TFE- d 2 solution containing 5 mM TCEP at 20°C and pH 7.5. a 1H Chemical shifts are reported in ppm with an accuracy of ±0.02 ppm. 13C Chemical shifts are reported in ppm with an accuracy of ±0.1 ppm. bCarbon chemical shifts first, and proton chemical shift in brackets. (DOC) [file pone.0072760.s008.doc]

**Table S3.**

| Residue | NH | ^13^C_α_ | ^1^H_α_ | ^13^C_β_ | ^1^H_β_ | Others^b^ |
| --- | --- | --- | --- | --- | --- | --- |
| **M1** |  | 55.5 | 3.70 | 34.2 | 1.94/1.88 | γ 30.9 (2.46/2.46), ε 15.7 (2.03 |
| **H2** | 7.77 | 56.2 | 4.53 | 30.2 | 3.12/3.04 |  |
| **G3** | 8.25 | 44.7 | 3.98/3.85 |  |  |  |
| **D4** | 8.10 | 53.8 | 4.68 | 40.7 | 2.70/2.70 |  |
| **T5** | 7.88 | 59.5 | 4.59 | 69.4 | 4.17 | δ_2_ 20.6 (1.28) |
| **P6** |  | 63.1 | 4.47 | 31.3 | 2.23/1.87 | γ 26.6 (1.95/2.00), δ 50.3 (3.78/3.68) |
| **T7** | 7.96 | 61.3 | 4.34 | 70.0 | 4.26 | δ_2_ 21.0 (1.21) |
| **L8** | 7.86 | 56.2 | 4.25 | 41.7 | 1.58/1.58 | γ 26.4 (1.58), δ_1_ 23.0 (0.83), δ_2_ 23.3 (0.88) |
| **H9** | 7.68 | 57.7 | 4.35 | 30.2 | 3.03/2.98 |  |
| **E10** | 8.02 | 58.5 | 3.94 | 29.0 | 1.99/1.94 | γ 36.2 (2.16/2.16) |
| **Y11** | 7.85 | 59.9 | 4.41 | 37.7 | 3.12/3.03 |  |
| **M12** | 7.86 | 56.4 | 4.24 | 31.9 | 2.06/2.06 | γ 31.8 (2.56/2.51), ε 15.8 (2.02) |
| **L13** | 7.60 | 55.8 | 4.17 | 41.6 | 1.72/1.53 | γ 26.4 (1.65), δ_1_ 22.5 (0.83), δ_2_ 24.1 (0.86) |
| **D14** | 7.73 | 54.2 | 4.60 | 40.6 | 2.70/2.61 |  |
| **L15** | 7.52 | 54.8 | 4.26 | 42.0 | 1.63/1.53 | γ 26.3 (1.59), δ_1_ 22.4 (0.80), δ_2_ 24.1 (0.85) |
| **Q16** | 7.85 | 53.2 | 4.60 | 28.5 | 2.16/1.98 | γ 33.0 (2.37/2.37) |
| **P17** |  | 63.3 | 4.38 | 31.4 | 2.25/1.90 | γ 26.7 (2.00/1.95), δ 50.0 (3.71/3.71) |
| **E18** | 8.71 | 56.9 | 4.29 | 29.4 | 2.08/1.98 | γ 35.8 (2.32/2.32) |
| **T19** | 7.93 | 62.2 | 4.30 | 69.0 | 4.30 | δ_2_ 21.0 (1.21) |
| **T20** | 7.79 | 62.9 | 4.22 | 69.0 | 4.26 | δ_2_ 20.9 (1.21) |
| **D21** | 8.02 | 55.1 | 4.55 | 40.3 | 2.68/2.68 |  |
| **L22** | 7.71 | 56.2 | 4.18 | 41.5 | 1.60/1.53 | γ 26.4 (1.53), δ_1_ 22.6 (0.80), δ_2_ 24.7 (0.88) |
| **Y23** | 7.82 | 59.0 | 4.39 | 37.8 | 3.08/3.01 |  |
| **C24** | 7.82 | 60.0 | 4.25 | 26.7 | 2.96/2.82 |  |
| **Y25** | 7.95 | 59.9 | 4.24 | 37.7 | 3.02/3.02 |  |
| **E26** | 8.25 | 58.0 | 4.04 | 29.1 | 2.03/2.03 | γ 35.8 (2.28/2.18) |
| **Q27** | 7.81 | 56.5 | 4.15 | 28.5 | 2.09/2.01 | γ 33.3 (2.13/2.13) |
| **L28** | 7.86 | 55.5 | 4.21 | 41.7 | 1.56/1.52 | γ 26.3 (1.68), δ_1_ 22.2 (0.82), δ_2_ 24.1 (0.87) |
| **N29** | 7.78 | 53.2 | 4.61 | 38.8 | 2.71/2.56 |  |
| **D30** | 7.96 | 54.0 | 4.65 | 40.9 | 2.69/2.69 |  |
| **S31** | 7.96 | 58.0 | 4.51 | 63.4 | 3.96/3.89 |  |
| **S32** | 8.23 | 58.4 | 4.45 | 63.4 | 3.95/3.89 |  |
| **E33** | 8.32 | 56.3 | 4.34 | 29.7 | 2.10/1.93 | γ 35.7 (2.30/2.30) |
| **E34** | 8.22 | 56.3 | 4.28 | 29.9 | 2.06/1.94 | γ 35.7 (2.28/2.28) |
| **E35** | 8.38 | 56.2 | 4.26 | 29.9 | 2.06/1.94 | γ 35.7 (2.30/2.30) |
| **D36** | 8.20 | 54.0 | 4.60 | 40.9 | 2.64/2.64 |  |
| **E37** | 8.20 | 56.2 | 4.29 | 29.9 | 2.06/1.94 | γ 35.8 (2.32/2.23) |
| **I38** | 7.98 | 61.2 | 4.10 | 38.3 | 1.87 | γ_1_ 26.9 (1.47/1.18), δ_1_ 12.1 (0.85), γ_2_ 16.6 (0.89) |
| **D39** | 8.19 | 53.9 | 4.58 | 40.5 | 2.72/2.56 |  |
| **G40** | 8.10 | 44.6 | 3.88/3.88 |  |  |  |
