# Supplementary material for: Conformational Dissection of a Viral Intrinsically Disordered Domain Involved in Cellular Transformation
Source: PLoS One. 2013 Sep 27;8(9):e72760. doi: 10.1371/journal.pone.0072760 (PMC3785498; doi:10.1371/journal.pone.0072760)
Supplement: Table S4 — 1JCαHα scalar coupling constant values (in Hz) measured for E7N. a The accuracy of the measurements is estimated to be ±0.5 Hz. bTFE-d2. c n.d.: not determined. (DOC) [file pone.0072760.s009.doc]

**Table S4.**

| Residue | water solution pH 5.00, 20°C | water solution pH 7.50, 4°C | TFE: water solution (1:1) pH 7.50, 20°C^b^ |
| --- | --- | --- | --- |
| **M1** | 149.9 | 145.6 | 147.7 |
| **H2** | 143.5 | 145.3 | 145.9 |
| **G3** | - | n.d. | - |
| **D4** | 145.2 | 143.5 | 144.9 |
| **T5** | 145.2 | 142.3 | 148.2 |
| **P6** | 148.5 | 149.0 | 148.6 |
| **T7** | 144.1 | 141.4 | 145.0 |
| **L8** | n.d.^c^ | 143.9 | 146.5 |
| **H9** | 144.6 | 145.3 | 148.8 |
| **E10** | 144.3 | 143.1 | 148.8 |
| **Y11** | 146.8 | 143.8 | 152.1 |
| **M12** | 145.9 | 143.5 | 148.2 |
| **L13** | n.d. | 145.4 | 146.6 |
| **D14** | 145.6 | 142.8 | 143.9 |
| **L15** | 143.8 | 142.7 | 140.7 |
| **Q16** | 143.4 | n.d. | n.d. |
| **P17** | 147.8 | 148.6 | 153.6 |
| **E18** | 143.6 | 144.0 | 143.5 |
| **T19** | 140.8 | 144.0 | 141.7 |
| **T20** | 141.7 | 143.9 | 144.5 |
| **D21** | n.d. | 142.4 | 146.6 |
| **L22** | 143.2 | 142.6 | 147.8 |
| **Y23** | 145.8 | 143.8 | 153.7 |
| **C24** | 145.4 | 144.8 | 148.3 |
| **Y25** | 145.7 | 147.3 | 149.4 |
| **E26** | n.d. | 145.6 | 151.6 |
| **Q27** | 142.8 | 145.2 | 144.0 |
| **L28** | n.d. | 143.2 | 147.0 |
| **N29** | 144.6 | 143.2 | n.d. |
| **D30** | 142.1 | 143.2 | 145.7 |
| **S31** | 142.4 | 142.2 | 142.4 |
| **S32** | 142.7 | 142.4 | 142.8 |
| **E33** | 144.1 | 145.9 | 146.1 |
| **E34** | n.d. | 146.3 | n.d. |
| **E35** | n.d. | n.d. | n.d. |
| **D36** | 144.1 | 147.7 | 146.1 |
| **E37** | n.d. | n.d. | 145.9 |
| **I38** | 144.4 | 144.5 | 142.4 |
| **D39** | n.d. | 142.8 | 141.9 |
| **G40** | - | - | - |
